# Large‐Scale Targeted Sequencing Study of Ischemic Stroke in the Han Chinese Population

**DOI:** 10.1161/JAHA.122.025245

**Published:** 2022-10-03

**Authors:** Mengyao Shi, Tanika N. Kelly, Zhengbao Zhu, Changwei Li, Chong Shen, Yingxian Sun, Aili Wang, Guangliang Shan, Xiaoqing Bu, Daoxia Guo, Jingbo Zhao, Tan Xu, Hao Peng, Tian Xu, Chongke Zhong, Xiao Sun, Jing Chen, Yonghong Zhang, Jiang He

**Affiliations:** ^1^ Department of Epidemiology Tulane University School of Public Health and Tropical Medicine New Orleans LA; ^2^ Tulane University Translational Science Institute New Orleans LA; ^3^ Department of Epidemiology School of Public Health, and Jiangsu Key Laboratory of Preventive and Translational Medicine for Geriatric Diseases Medical College of Soochow University Suzhou China; ^4^ Department of Epidemiology, School of Public Health Nanjing Medical University Nanjing China; ^5^ Department of Cardiology the First Affiliated Hospital of China Medical University Shenyang China; ^6^ Department of Epidemiology, School of Basic Medicine Peking Union Medical College Beijing China; ^7^ Department of Epidemiology, School of Public Health and Management Chongqing Medical University Chongqing China; ^8^ Department of Epidemiology, School of Public Health Harbin Medical University Harbin China; ^9^ Department of Neurology Affiliated Hospital of Nantong University Nantong China

**Keywords:** gene–environment interaction, genetic analysis, ischemic stroke, targeted sequencing study, Ischemic Stroke, Genetics

## Abstract

**Background:**

Ischemic stroke is likely caused by interactions of multiple genes and environmental determinants. However, large‐scale sequencing studies to discern functional genetic variants and their interactions with clinical and lifestyle risk factors on ischemic stroke are limited.

**Methods and Results:**

We sequenced functional regions of 740 previously identified genes associated with atherosclerotic disease among 999 ischemic stroke cases and 1001 controls of Chinese ancestry. Multiple logistic regression models were used to examine the associations between variants and ischemic stroke and test interactions between variants and clinical and lifestyle risk factors. Functional variants achieving suggestive significance were replicated in an independent sample of 4724 ischemic stroke cases and 5029 controls. Driven by variant main effects, each minor allele of the correlated rs174535, rs174545, and rs3834458 variants at *MYRF‐FADS1‐FADS2* conferred an average 0.83‐fold (95% CI, 0.78–0.88) decreased odds of stroke. Significant main effects of *MTHFR* rs1801133 missense variant were also observed, with each copy of the A allele associated with a 1.20‐fold (95% CI, 1.13–1.27) higher odds of ischemic stroke. The functional *ALDH2* rs671 variant was identified in interaction analyses with alcohol drinking (*Meta‐P*=3.39×10^−17^). Each minor allele conferred a 0.54‐fold (95% CI, 0.45–0.64) decreased odds of stroke among drinkers and a 0.89‐fold (95% CI, 0.83–0.97) decreased odds among nondrinkers.

**Conclusions:**

Significant associations at *MYRF‐FADS1‐FADS2* indicate that genetically elevated polyunsaturated fatty acids may decrease ischemic stroke risk in East Asians. Significant associations at *MTHFR* and *ALDH2* robustly confirm deleterious effects of genetically elevated homocysteine and alcohol intake, respectively, on ischemic stroke.

**Nonstandard Abbreviations and Acronyms**
CATIS: China Antihypertensive Trial in Acute Ischemic Stroke
df: degree‐of‐freedom
GWAS: genome‐wide association study
IIPAIS: Infectious Factors, Inflammatory Markers and Prognosis of Acute Ischemic Stroke
indel: insertion/deletion

**Clinical Perspective**

**What Is New?**

- We identified 3 correlated variants (rs174535, rs174545, and rs3834458) at *MYRF‐FADS1‐FADS2* that inversely associated with ischemic stroke, and we provide additional evidence for associations between *MTHFR* rs1801133 and ischemic stroke, as well as interactions of *ALDH2* rs671 with alcohol consumption on risk of this complex phenotype.

**What Are the Clinical Implications?**

- Our findings at the *MYRF‐FADS1‐FADS2* locus provide the first causal evidence for a protective role of genetically elevated polyunsaturated fatty acids in ischemic stroke among individuals of East Asian ancestry.
- Observed associations of *MTHFR* rs1801133 and *ALDH2* rs671 with ischemic stroke provide support for homocysteine and alcohol, respectively, as important mechanisms in ischemic stroke and these findings support guidance to reduce homocysteine levels and limit alcohol consumption for the prevention of stroke.

Stroke is the third‐leading cause of death and disability‐adjusted life‐years globally.^1^, ^2^ China has the highest stroke morbidity and mortality in the world, with >2.4 million incident stroke cases and 1.1 million stroke deaths annually.^3^, ^4^, ^5^ Ischemic stroke represents the most common stroke subtype, comprising 87% of total stroke cases.^6^ As a complex phenotype, ischemic stroke is likely caused by both genetic and environmental determinants, as well as their interactions.^7^ Candidate genes studies and genome‐wide association studies (GWASs) have made important strides in identifying independent loci associated with ischemic stroke (Table S1).^8^, ^9^ Despite such advancements, the causal genes and variants at these loci remain largely unknown. In addition, there is a paucity of research examining interactions of these loci with known clinical and lifestyle risk factors for stroke.^10^, ^11^, ^12^, ^13^ Large‐scale sequencing studies could help to refine GWAS signals, providing novel targets for the development of molecular‐based therapies for stroke prevention and treatment. Furthermore, discerning environmental modifiers of gene–stroke associations could identify high‐risk subgroups who might be specially benefited through known therapies or lifestyle modifications.

To identify genes and variants for stroke and related atherosclerosis phenotypes, we conducted a systematic literature search for published GWASs and candidate gene studies. In total, 740 genes from 407 previously identified loci were reported, and we conducted a targeted sequencing study among 999 ischemic stroke cases and 1001 healthy controls of Han Chinese ancestry. Our discovery‐stage analyses assessed the main effects of sequenced variants, as well as their interactions with clinical and lifestyle risk factors, on stroke. Findings were replicated among an independent sample of 9753 Han Chinese participants, including 4724 ischemic stroke cases and 5029 controls. For the current study, we aimed to identify functional genetic variants for stroke as well as functional genetic variants that may interact with clinical and lifestyle risk factors to influence ischemic stroke risk. Such findings could provide novel insights into the underlying biological mechanisms of stroke and also inform the development of preventive strategies for stroke in genetically high‐risk subgroups.

## Methods

The data that support the findings of this study are available from the corresponding author upon reasonable request.

### Study Participants

A total of 5723 ischemic stroke cases and 6030 geography‐matched controls from China were included in the current study. Because environmental and clinical risk factors vary substantially by geography (Table S2), the controls were frequency matched to cases based on regions of China. For our study, regions included Middle China (including Anhui, Jiangsu, Henan, and Shandong provinces) and North China (including Hebei, Ningxia, Liaoning, Jilin, Inner Mongolia, and Heilongjiang provinces). Cases were recruited from the CATIS (China Antihypertensive Trial in Acute Ischemic Stroke) study (n=2652),^14^ the IIPAIS (Infectious Factors, Inflammatory Markers and Prognosis of Acute Ischemic Stroke) study (n=890),^15^ and community‐based epidemiology studies in Jiangsu (n=2074) and Harbin (n=107).^16^ Geography‐matched controls were recruited from community‐based health surveys including the China National Health Survey (n=318),^17^ Northeast China Rural Cardiovascular Health Study (n=1312),^18^ and other community‐based epidemiology studies in Jiangsu (n=3665), and Harbin (n=735).^16^, ^19^ The discovery cohort was composed of 999 patients with ischemic stroke from the CATIS study with the earliest ages of stroke onset. This strategy was used to enrich the discovery sample for disease‐causing variants. A total of 1001 healthy controls who were older and had fewer stroke risk factors (eg, lower body mass index, lower blood pressure, lower blood lipid levels, and lower frequency of hypertension, diabetes, and hyperlipidemia) were also selected. The remaining samples were used in the replication study. The study was approved by the Institutional Review Boards at Tulane University in the United States and the Ethical Committees at Soochow University and other participating institutes in China. Written informed consent was obtained from all study participants.

Ischemic stroke was defined as an episode of neurological dysfunction caused by focal cerebral, spinal, or retinal infarction,^20^ which was confirmed in all patients by computed tomography or magnetic resonance imaging. Controls were judged to be free of atherosclerotic diseases (eg, ischemic stroke, transient ischemic attack, myocardial infarction, and peripheral arterial disease) on the basis of medical history and clinical examination.

### Targeted Gene Sequencing and Variant Genotyping

Atherosclerosis is an established risk factor for ischemic stroke.^21^ To maximize the number of genes sequenced, we included genes and variants for both stroke and related atherosclerosis phenotypes (coronary heart disease, myocardial infarction, carotid intima‐media thickness, and arterial aneurysm). We conducted a systematic search of MEDLINE, the GWAS catalog,^22^ and the HUGE Navigator phenopedia^23^ for GWAS and candidate gene studies published between January 1996 and November 2014. Nominal significance was employed for candidate gene studies, while a genome‐wide significance level (5×10^−8^) was employed for GWAS. At identified GWAS loci, nearby genes for each variant were selected. In addition, expression quantitative trait loci databases were queried to select genes with differential expression according to the reported variant or a variant in linkage disequilibrium with the reported variant. The systematic search and database query identified a total of 740 genes from 407 one‐megabase loci for targeted gene‐sequencing study.

Among the 2000 discovery‐stage participants, functional regions of the 740 identified genes, including promoters, 5′‐untranslated region, exons, splice sites/junctions, and 3'‐untranslated region, were sequenced on the Illumina HiSeq 4000 system (Illumina Inc., San Diego, CA) with custom capture using the NimbleGen SeqCap EZ Choice probes (Roche, Basel, Switzerland). The average depth and breadth of coverage was 140× and 99.4%, respectively. A total of 82 358 single‐nucleotide polymorphisms (SNPs) and 8922 insertion/deletions (indels) were identified through sequencing. Some 81 365 SNPs and 8464 indels passed our stringent quality control protocol, which filtered 548 SNPs with missing rate >10%, 70 SNPs with differential missingness by case and control status (*P*<1.0×10^−5^), and 375 SNPs and 458 indels with Hardy–Weinberg equilibrium *P*<1.0×10^−5^ among control. Variants with minor allele count <10, including 72 524 SNPs and 7160 indels, were additionally excluded from the single variant analyses, leaving 8841 SNPs and 1304 indels for this analysis.

Among the 9753 replication stage participants, we used the SNPscan system (Genesky Biotechnologies Inc., Shanghai, China) to carry out genotyping of 48 functional variants (45 SNPs and 3 indels) identified in the discovery stage. A total of 34 variants (31 SNPs and 3 indels) were successfully genotyped. After excluding variants with missing rate >10%, minor allele count <10, or Hardy–Weinberg equilibrium *P*<1.0×10^−2^ in the controls, 31 SNPs and 3 indels remained for the analysis in the replication stage.

### Measurement of Demographic, Lifestyle, and Clinical Variables

Data on demographic, lifestyle, medical history, and clinical variables were collected in cases and controls across studies using similar protocols and harmonized for our analysis. Demographic characteristics (age and sex), lifestyle risk factors (current drinking and current smoking), medical history (history of hypertension, hyperlipidemia, diabetes, and atherosclerotic disease), and medication use (antihypertensive medication, lipid‐lowing medication, and hypoglycemic medication) were collected by trained staff using standard questionnaires. Blood pressure was estimated as the average of 3 blood pressure measurements obtained by trained nurses according to a common protocol adapted from procedures recommended by the American Heart Association.^24^ Blood pressure was measured with a standard mercury sphygmomanometer and appropriate cuff size based on participant's arm circumference. Body weight and height were measured using a regularly calibrated stadiometer and balance‐beam scale with patients wearing light clothing and no shoes. Body mass index was calculated as weight in kilograms divided by height in meters squared. Serum lipids and plasma glucose were measured after at least 8 hours of fasting at local clinical laboratories that participated in a national standardization program.

### Statistical Analysis

Characteristics of study participants were presented as means and SDs for continuous variables and as numbers and percentages for categorical variables. Before association analyses, triglyceride values were log‐transformed. In the discovery stage, multiple logistic regression models were used to examine the main effect of each variant on ischemic stroke. Interactions of each variant with demographic (sex), lifestyle (alcohol drinking and cigarette smoking) and clinical risk factors (body mass index, blood pressure, lipids, glucose, obesity, history of hypertension, and history of dyslipidemia) were assessed. The variant by risk factor interaction term as well as variant and risk factor were included in the logistic regression model. One degree‐of‐freedom (df) interaction and 2‐df joint main effects and interaction tests were performed,^25^, ^26^ with the 2‐df joint test used to maximize power to detect variants with both moderate main effects and moderate interaction effects.^25^ All models were adjusted for the fixed effects of age, sex, province and the first 4 ancestry principal components.^27^ Functional SNPs and indels achieving *P*<1.0×10^−4^ in any of the analyses were genotyped and tested in main effects, and 1‐df and 2‐df interaction analyses among replication study participants. Aggregate rare variant analyses were also performed. Rare variants were grouped by gene and tested for association with stroke using the sequence kernel association test, employing the same covariable adjustments as in the single marker analyses.^28^ Gene‐based signals achieving *P*<1.0×10^−4^ in the aggregate rare variant analyses were also moved forward for replication study. Statistical analyses were performed with PLINK version 1.9 and SAS version 9.4 (SAS Institute, Cary, NC).

Replication stage analyses employed the same multiple logistic regression models that were used in the discovery stage. Standard inverse variance weighted meta‐analysis was used to combine results from each of the main effects and 1‐df interaction analyses across the discovery and replication stages. For the 2‐df joint tests, meta‐analysis was conducted using methods developed by Manning et al.^29^ All meta‐analyses were conducted using METAL software (version 2010‐02‐08).^30^ Variants achieving nominal significance in the replication stage and a Bonferroni‐corrected meta‐analysis *P* value <5.0×10^−6^ were considered statistically significant.

All of the analyses above were conducted under the assumption of an additive genetic model. For identified variants, sensitivity analyses assuming dominant and recessive genetic models were conducted. In addition, for variants identified in 1‐df interaction or 2‐df joint tests, sensitivity analyses using robust standard errors were conducted to control for possible inflation. Furthermore, given large differences in patterns of alcohol drinking between Chinese men and women,^17^ significant variant‐alcohol drinking interactions were further explored in sex‐stratified analyses.

## Results

Characteristics of the discovery and replication study participants are presented according to case and control status in Table 1. In the discovery and replication stages, ischemic stroke cases were more likely to be men and have a history of hypertension, hyperlipidemia, diabetes, and medication use. On average, patients with ischemic stroke had higher systolic and diastolic blood pressure, body mass index, serum triglycerides, and fasting plasma glucose. Because of selection, ischemic stroke cases were younger than healthy controls in discovery samples. Ischemic stroke cases were less likely to drink alcohol in discovery and replication samples and smoke cigarettes in replication samples.

**Table 1:** Characteristics of Ischemic Stroke Cases and Controls at Discovery and Replication Stages

Characteristics*	Discovery study			Replication study		
	Cases (n=999)	Controls (n=1001)	*P* value	Cases (n=4724)	Controls (n=5029)	*P* value
Age, y	62.4±10.4	65.4±6.4	<0.001	64.3±10.9	63.4±9.1	<0.001
Male, n (%)	622 (62.3)	520 (52.0)	<0.001	2974 (63.0)	2439 (48.5)	<0.001
Current cigarette smoking, n (%)	368 (36.8)	356 (35.6)	0.554	1528 (32.4)	1842 (36.8)	<0.001
Alcohol drinking, n (%)	287 (28.7)	476 (47.6)	<0.001	1175 (25.2)	1575 (31.8)	<0.001
Systolic blood pressure, mm Hg	167.2±16.7	127.5±12.4	<0.001	155.7±21.9	132.6±15.0	<0.001
Diastolic blood pressure, mm Hg	96.4±10.4	75.4±8.0	<0.001	91.2±12.8	80.9±9.2	<0.001
Body mass index, kg/m^2^	25.1±3.3	23.6±3.4	<0.001	24.7±3.2	24.1±3.4	<0.001
Total cholesterol, mg/dL	197.2±45.2	206.1±40.6	<0.001	189.1±47.2	194.1±41.4	<0.001
Triglycerides, mg/dL	155.0±107.2	131.1±70.9	<0.001	160.3±135.5	152.3±131.1	0.002
Low‐density lipoprotein cholesterol, mg/dL	114.5±39.1	114.8±36.0	0.934	112.5±38.7	113.7±45.6	0.275
High‐density lipoprotein cholesterol, mg/dL	50.7±17.0	55.7±13.9	<0.001	47.6±17.4	58.0±37.5	<0.001
Fasting plasma glucose, mg/dL	123.4±52.8	100.5±19.1	<0.001	119.6±51.5	101.3±27.4	<0.001
Obesity,† n (%)	153 (17.5)	95 (9.6)	<0.001	341 (13.2)	595 (11.9)	0.093
History of hypertension, n (%)	793 (79.4)	113 (11.3)	<0.001	3316 (71.8)	1302 (26.0)	<0.001
History of hyperlipidemia, n (%)	60 (6.0)	24 (2.4)	<0.001	397 (8.9)	114 (3.5)	<0.001
History of diabetes, n (%)	187 (18.7)	0 (0.0)	<0.001	1025 (22.0)	436 (8.7)	<0.001
Use of antihypertensive medications, n (%)	479 (48.0)	0 (0.0)	<0.001	1661 (56.2)	1029 (36.5)	<0.001
Use of lipid‐lowering medications, n (%)	28 (2.8)	8 (0.8)	0.001	141 (3.0)	25 (0.5)	<0.001
Use of hypoglycemic medications, n (%)	76 (7.6)	0 (0.0)	<0.001	503 (10.7)	66 (1.3)	<0.001

* Continuous variables are expressed as mean±SD. Categorical variables are expressed as number (percentage).

^†^ Obesity was defined as body mass index ≥28 kg/m^2^.

**Figure 1:**
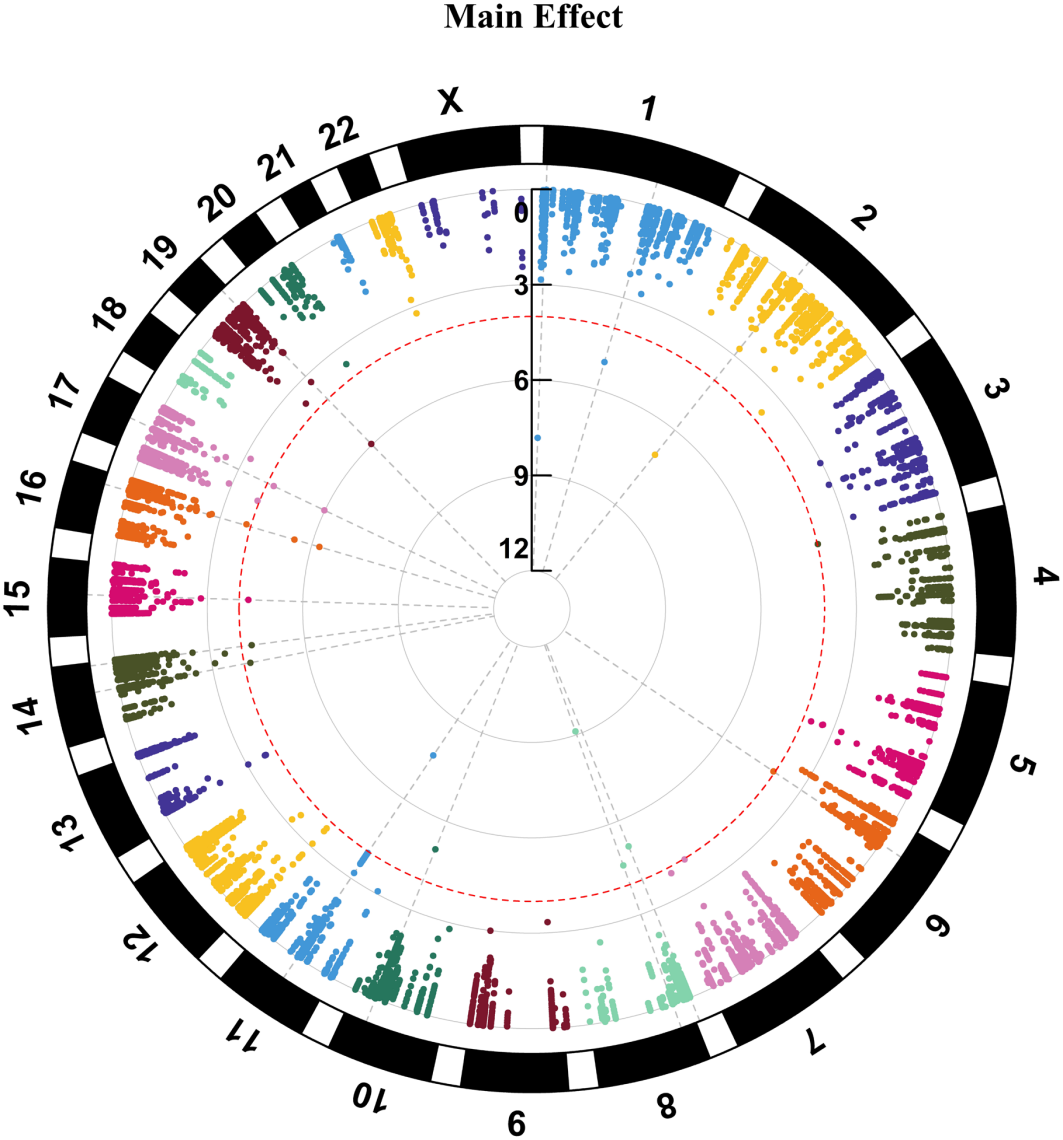
Circular Manhattan plot displaying variants achieving suggestive significance in the discovery‐stage main‐effects analyses. Red dashed lines indicate suggestive significance (*P*<1.0×10^−4^) in the discovery stage.

**Figure 2:**
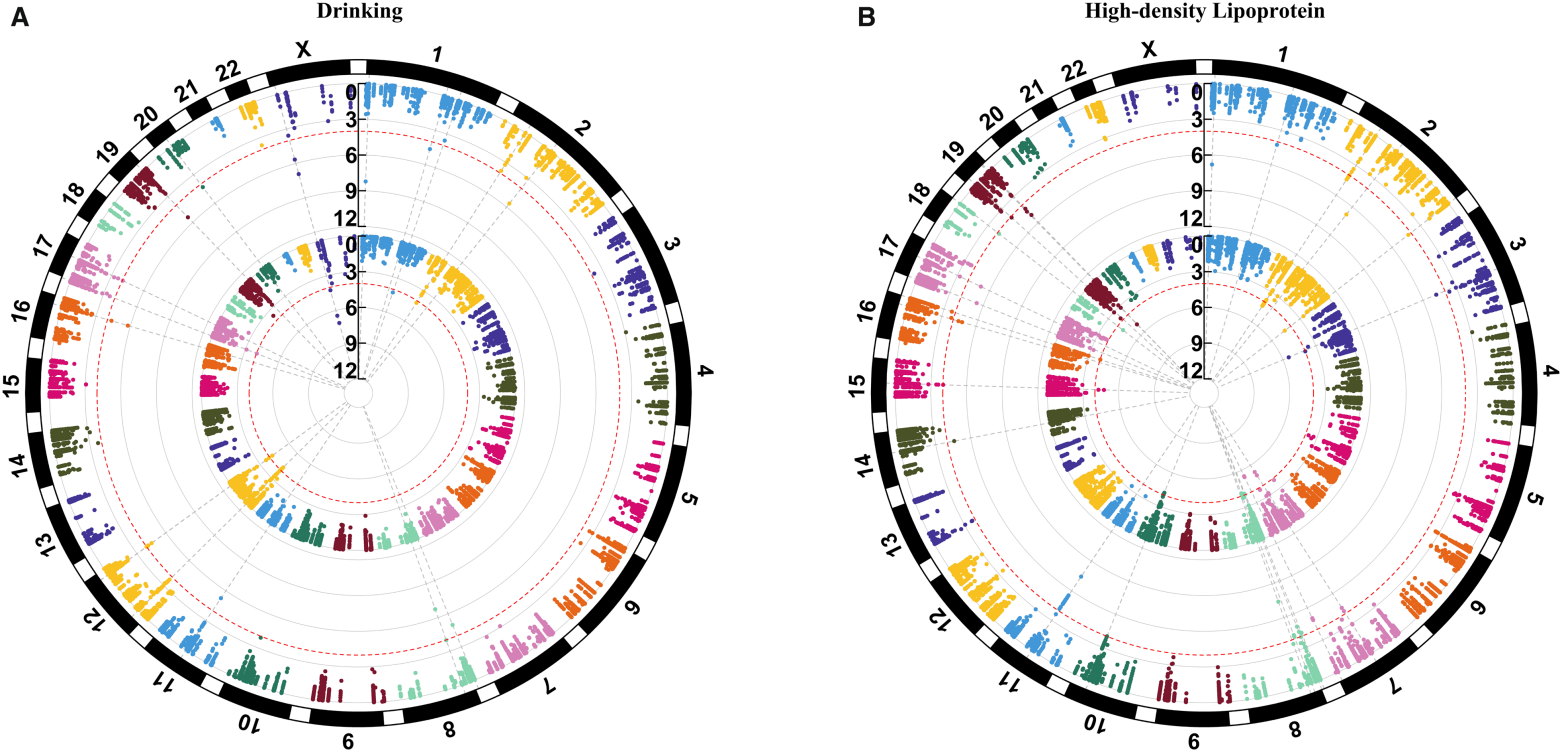
Circular Manhattan plots displaying variants achieving suggestive significance in the 1‐df interaction (inner circle) and 2‐df joint tests (outer circle). **A**, Interaction with alcohol drinking; (**B**) interaction with high‐density lipoprotein cholesterol. Red dashed lines indicate suggestive significance (*P*<1.0×10^−4^) in the discovery stage.

Discovery‐stage analyses revealed 112 variants suggestively associated with ischemic stroke (*P*<1.0×10^−4^), including 85 SNPs and 27 indels (Figures 1 and 2, Figures S1 and S2, and Table S3 through S14). Seventeen variants were suggestively associated with stroke in the main‐effects analyses (Table S3). In the 1‐df interaction tests, 59 variants were suggestively identified for ischemic stroke, including 10 variants that interacted with alcohol drinking, 5 that interacted with smoking, 1 that interacted with low‐density lipoprotein cholesterol, 31 that interacted with high‐density lipoprotein (HDL) cholesterol, 13 that interacted with fasting plasma glucose, and 2 that interacted with history of hypertension (Tables S3 through S14). For the 2‐df joint tests, a total of 85 variants were suggestively identified for ischemic stroke. These included 13 variants associated jointly with sex, 17 variants associated jointly with alcohol drinking, 16 associated jointly with smoking, 13 associated jointly with low‐density lipoprotein cholesterol, 59 associated jointly with HDL‐cholesterol, 16 associated jointly with fasting plasma glucose, 5 associated jointly with history of hyperlipidemia, nine variants associated jointly with history of hypertension, seven variants associated jointly with body mass index, 15 associated jointly with triglycerides, and 10 associated jointly with obesity (Tables S3 through S14). Aggregate rare variant analyses did not identify any genes suggestively associated with stroke (all *P*>1.0×10^−4^; data not shown).

**Figure 3:**
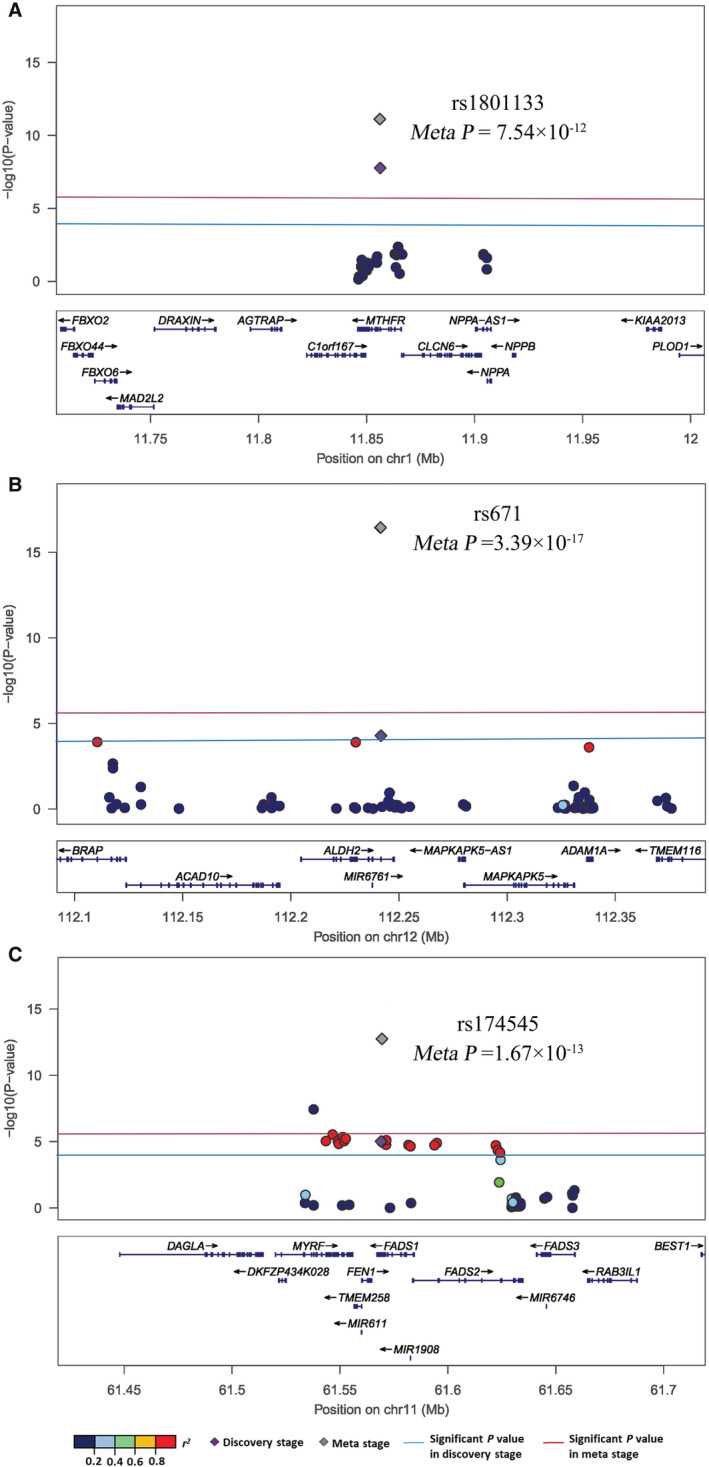
Regional association plots for significant stroke signals. **A**, Main effects analysis of rs1801133; **B**, 1‐df analysis of rs671 with alcohol drinking; C: 2‐df analysis of rs174545 with high‐density lipoprotein cholesterol.

Among the 112 variants identified in the discovery stage, 48 exonic variants (45 SNPs and 3 indels) were selected for replication study. Among them, 31 SNPs and 3 indels were successfully genotyped and further tested in the replication and meta‐analyses (Tables S15 and S16). One suggestive signal from the discovery stage main effects analysis, *MTHFR* rs1801133, was replicated and achieved *P*=7.5×10^−12^ in meta‐analyses of results from the discovery and replication studies (Table 2; Figure 3A). Each copy of the A allele conferred 1.20‐fold (95% CI, 1.13–1.27) higher odds of ischemic stroke in meta‐analyses.

**Table 2:** Variants Achieving Significance in Main Effects Meta‐Analyses (*P*<5×10^−6^)

Variant	Chr	Position (Build 37)	Gene	CA/OA	CAF	Stage	Odds ratios	95% CI	*P* value
rs1801133	1	11 856 378	*MTHFR*	A/G	0.55	Discovery	1.45	(1.26, 1.66)	1.54E‐08
					0.48	Replication	1.16	(1.10, 1.23)	6.56E‐07
						Meta‐analysis	1.20	(1.13, 1.27)	7.54E‐12
rs174535*	11	61 551 356	*MYRF*	C/T	0.34	Discovery	0.76	(0.67, 0.88)	1.35E‐04
					0.37	Replication	0.84	(0.80, 0.89)	3.61E‐08
						Meta‐analysis	0.84	(0.79, 0.89)	4.81E‐11
rs174545*	11	61 569 306	*FADS1*	G/C	0.33	Discovery	0.76	(0.67, 0.88)	1.48E‐04
					0.37	Replication	0.84	(0.80, 0.89)	1.98E‐08
						Meta‐analysis	0.83	(0.78, 0.88)	2.78E‐11
rs3834458*	11	61 594 920	*FADS2*	C/CT	0.33	Discovery	0.76	(0.67, 0.88)	1.85E‐04
					0.36	Replication	0.84	(0.80, 0.89)	1.45E‐08
						Meta‐analysis	0.83	(0.78, 0.88)	2.30E‐11
rs3782886†	12	112 110 489	*BRAP*	C/T	0.16	Discovery	0.91	(0.77, 1.09)	2.88E‐01
					0.20	Replication	0.82	(0.76, 0.89)	1.15E‐07
						Meta‐analysis	0.84	(0.79, 0.89)	1.22E‐07
rs671†	12	112 241 766	*ALDH2*	A/G	0.16	Discovery	0.90	(0.76, 1.08)	2.48E‐01
					0.19	Replication	0.81	(0.75, 0.88)	2.44E‐08
						Meta‐analysis	0.83	(0.78, 0.88)	2.36E‐08
rs78069066†	12	112 337 924	*ADAM1A*	A/G	0.16	Discovery	0.90	(0.76, 1.08)	2.38E‐01
					0.20	Replication	0.83	(0.76, 0.89)	1.60E‐07
						Meta‐analysis	0.84	(0.79, 0.89)	1.28E‐07

Based on an additive genetic model, adjusting for age, sex, province, and the first 4 genetic principal components. CA indicates coded allele; CAF, coded allele frequency; Chr, chromosome; OA, other allele; and OR, odds ratio.

* rs174535, rs174545, and rs3834458 were highly correlated, smallest *R* ^2^=0.99.

^†^ rs3782886, rs671, and rs78069066 were highly correlated, smallest *R* ^2^=0.98.

Among the functional variants that achieved suggestive significance in the discovery‐stage 1‐df tests, the interactions of alcohol with rs3782886 at *BRAP*, rs671 at *ALDH2*, and rs78069066 at *ADAM1A* (3 correlated variants with all pairwise *r* ^2^≥0.98) were replicated and achieved *P*=1.3×10^−15^, *P*=3.4×10^−17^, and *P*=1.6×10^−15^, respectively, in the meta‐analysis of discovery and replication stage findings (Table 3; Figure 3B). In meta‐analysis, each copy of the rs671 A allele was associated with a 0.54‐fold (95% CI, 0.45–0.64) decreased risk of ischemic stroke among drinkers and an attenuated 0.89‐fold (95% CI, 0.83–0.97) decreased risk of ischemic stroke among nondrinkers. When further stratified by sex, protective effects were observed among male but not female participants (Tables S17).

**Table 3:** Variants Achieving Significance in 1‐df Interaction Test Meta‐Analyses (*P*<5×10^−6^)

Variant	Chr	Position (Build 37)	Gene	CA/OA	CAF	Stage	Drinkers			Nondrinkers			1‐df interaction *P* value
							OR	95% CI	*P* value	OR	95% CI	*P* value	
rs3782886*	12	112 110 489	*BRAP*	C/T	0.16	Discovery	0.37	(0.24, 0.59)	2.06E‐05	1.01	(0.82, 1.23)	9.60E‐01	1.19E‐04
					0.20	Replication	0.61	(0.51, 0.74)	1.93E‐07	0.88	(0.81, 0.96)	2.68E‐03	1.72E‐12
						Meta	0.57	(0.48, 0.68)	1.25E‐10	0.90	(0.83, 0.97)	5.96E‐03	1.33E‐15
rs671*	12	112 241 766	*ALDH2*	A/G	0.16	Discovery	0.34	(0.21, 0.54)	6.47E‐06	1.01	(0.83, 1.24)	9.02E‐01	5.47E‐05
					0.19	Replication	0.58	(0.48, 0.70)	1.39E‐08	0.88	(0.80, 0.95)	2.27E‐03	9.20E‐14
						Meta	0.54	(0.45, 0.64)	3.55E‐12	0.89	(0.83, 0.97)	5.60E‐03	3.39E‐17
rs78069066*	12	112 337 924	*ADAM1A*	A/G	0.16	Discovery	0.39	(0.25, 0.62)	4.36E‐05	0.99	(0.81, 1.22)	9.42E‐01	2.45E‐04
					0.20	Replication	0.61	(0.50, 0.73)	1.14E‐07	0.88	(0.81, 0.96)	3.19E‐03	1.20E‐12
						Meta	0.57	(0.48, 0.67)	1.01E‐10	0.90	(0.83, 0.97)	5.99E‐03	1.55E‐15

Based on an additive genetic model, adjusting for age, sex, province, and the first 4 genetic principal components. CA indicates coded allele; CAF, coded allele frequency; Chr, chromosome; OA, other allele; and OR, odds ratio.

* rs3782886, rs671, and rs78069066 were highly correlated, smallest *R* ^2^=0.98.

Among those variants identified in the discovery‐stage 2–df joint test, 7 variants from 3 loci—*MTHFR*, *BRAP‐ALDH2‐ADAM1A*, and *MYRF‐FADS1‐FADS2*—were replicated and achieved Bonferroni significance in meta‐analyses (Table S16). *MTHFR* variant rs1801133 demonstrated significant 2‐df joint effects with each of sex, body mass index, fasting plasma glucose, HDL cholesterol, low‐density lipoprotein cholesterol, triglycerides, drinking, smoking, obesity, and history of hyperlipidemia on stroke (*Meta‐P*=2.7×10^−11^, 3.9×10^−17^, 1.1×10^−15^, 1.2×10^−11^, 7.5×10^−12^, 2.3×10^−12^, 2.5×10^−12^, 7.0×10^−12^, 8.4×10^−17^, and 7.2×10^−9^, respectively). The results of the 2‐df test were driven by the strong main effects of the *MTHFR* variant (Table 2), with findings from the 1‐df tests generally nonsignificant across all risk factors (Table S16). In addition, the *BRAP‐ALDH2‐ADAM1A* variants rs3782886, rs671, and rs78069066 achieved meta‐analysis *P*=2.5×10^−23^, 2.6×10^−25^, and 2.7×10^−23^, respectively, in the 2‐df joint tests with alcohol drinking. These variants displayed significant main effects (Table 2), as well as significant 1‐df interactions with alcohol drinking (Table 3) in meta‐analyses. At the *MYRF‐FADS1‐FADS2* locus, correlated variants rs174535, rs174545, and rs3834458 (all pairwise *r* ^2^≥0.99) were identified for stroke in 2‐df joint tests with HDL cholesterol (*Meta‐P*=1.2×10^−12^, 1.7×10^−13^, and 1.7×10^−12^, respectively; Figure 3C). Significant associations of rs174535, rs174545, and rs3834458 were also observed in meta‐analyses of main effects (*Meta‐P*=4.8×10^−11^, 2.8×10^−11^, and 2.3×10^−11^, respectively; Table 2).

Sensitivity analyses employing dominant and recessive genetic models demonstrated results consistent with the primary analyses, which assumed an additive model (Tables S18 through S19). Similarly, results of sensitivity analyses using robust standard errors for variants identified using the 1‐df interaction or 2‐df joint tests were also consistent with those of the primary analysis (Tables S20 and S21).

## Discussion

Our large‐scale targeted sequencing study identified 3 independent loci associated with ischemic stroke in a combined sample of 11 753 Han Chinese participants. Three correlated ischemic stroke variants (rs174535, rs174545, and rs3834458) at *MYRF‐FADS1‐FADS2* were identified for the first time in East Asian participants. Discovered in 2‐df joint tests with HDL‐cholesterol, each minor allele conferred an average 0.8‐fold decreased odds of stroke. Conversely, the well‐known *MTHFR* rs1801133 missense variant was associated with a significant 1.2‐fold increased odds of stroke in our main effects meta‐analysis. At *BRAP*‐*ALDH2*‐*ADAM1A*, 3 correlated variants (rs3782886, rs671, and rs78069066) were identified in the 1‐df interaction tests with alcohol drinking. These variants, likely reflecting the association of the functional *ALDH2* rs671 missense variant, conferred 0.54‐fold decreased odds of ischemic stroke among drinkers in meta‐analyses, with substantially attenuated associations among nondrinkers. In total, our findings provide novel functional insights into biological mechanisms underlying ischemic stroke.

The 3 correlated variants (rs174535, rs174545, and rs3834458) identified at *MYRF‐FADS1‐FADS2* appeared driven predominantly by variant main effects. Based on the most recent genome build, rs174545 lies in the 3′‐untranslated region of *FADS1*, and rs3834458 is in an upstream region of *FADS2*. In contrast, *MYRF* variant rs174535 is exonic, with missense functionality in some isoforms. Despite the functional potential of the *MYRF* variant on the protein it encodes, previous studies have identified associations of these variants (or their linkage disequilibrium proxies) with concentrations of omega‐6 polyunsaturated fatty acids (PUFAs).^31^, ^32^, ^33^, ^34^, ^35^ Omega‐6 PUFAs are regulated by the *FADS1* and *FADS2* genes,^36^, ^37^, ^38^ suggesting that functional variants in these genes may underlie the observed associations. Similar to our findings, the minor allele of *FADS1* rs174547, a SNP in high linkage disequilibrium with the 3 identified variants, was associated with lower odds of stroke in a previous Mendelian randomization study in a predominantly European population.^39^ Previous observational studies have provided evidence of the protective roles of PUFAs on stroke, and a clinical trial further showed that diets with higher PUFAs significantly lowered the incidence of stroke.^38^, ^40^, ^41^ The anti‐inflammatory and anticoagulant characteristics of PUFAs have been suggested as potential mechanisms of action for their beneficial effects. Our findings provide the first causal evidence for a protective role of genetically elevated PUFAs in ischemic stroke among individuals of East Asian ancestry.

The *MTHFR* rs1801133 variant strongly associated with ischemic stroke in our main‐effects analysis and drove the significant findings of several 2‐df joint tests with clinical and lifestyle risk factors. *MTHFR* encodes the methylenetetrahydrofolate reductase enzyme, which plays a critical role in converting homocysteine to methionine. Substitution of an alanine amino acid with valine at the 222nd residue, which is encoded by rs1801133, results in a more thermolabile protein and causes reduced enzymatic function and increased levels of homocysteine.^42^, ^43^ Elevated plasma homocysteine has been associated with stroke previously by observational studies.^43^, ^44^, ^45^, ^46^ Homocysteine may exert its effect by decreasing nitric oxide generation, inducing oxidative injury and increased platelet adhesion.^47^, ^48^ Homocysteine can be lowered by folic acid supplementation, and several clinical trials have demonstrated that homocysteine lowering via folic acid supplementation moderately reduces stroke risk.^49^ Associations of homocysteine‐regulating genetic variants, which are not confounded by traditional risk factors, could support its potential causal relationship with ischemic stroke. Indeed, rs1801133 has been associated with stroke in previous studies.^42^, ^50^, ^51^, ^52^ However, these studies have had relatively small sample sizes and used permissive statistical significance thresholds. We are the first to report this SNP in a large‐scale study at a level surpassing genome‐wide significance. The successful replication of this finding further implicates the potential causal role of homocysteine in stroke.

Three variants at *BRAP‐ALDH2‐ADAM1A* (rs3782886, rs671, and rs78069066), which interacted with alcohol drinking, are specific to populations of Asian ancestry. Among drinkers, each copy of the minor alleles of these variants associated with a 0.5‐fold decreased odds of ischemic stroke, a finding that was substantially attenuated among nondrinkers. The strong effect observed in drinkers resulted in a more attenuated but still genome‐wide significant signal of this variant in the entire sample. Since the nondrinker group included former drinkers, we observed nominal associations within this subgroup. At the identified locus, *ALDH2* encodes an enzyme essential to alcohol metabolism. The well‐known rs671 missense SNP results in a glutamic acid to lysine amino acid substitution at the 504th residue, decreasing activity of the encoded aldehyde dehydrogenase 2 enzyme. Decreased enzymatic activity allows acetaldehyde to accumulate,^53^, ^54^ resulting in reduced tolerance to alcohol and, likewise, reduced alcohol intake.^53^, ^54^ Given the important role of rs671 in alcohol metabolism, this SNP likely represents the causal variant underlying the signal observed at this locus and a genomic proxy for alcohol intake. Since drinkers carrying the rs671 variant are likely to consume less alcohol compared with noncarriers, these data suggest that decreased alcohol intake reduces ischemic stroke risk. Similar to our findings, previous studies have identified an association of rs671 with ischemic stroke,^55^, ^56^, ^57^ including a recent study of 121 698 Chinese participants that demonstrated a positive log‐linear relationship of genetically predicted alcohol intake with ischemic stroke.^58^ Interestingly, when further stratified the population by sex, significant effects were only observed among male participants. These findings are consistent with those of Millwood and his colleagues^58^ and likely relates to more modest alcohol drinking in women,^59^ even within the “drinker” subgroup. Previous observational studies have supported an inverse association between low to moderate alcohol consumption and stroke risk.^60^ Our findings, together with results from Millwood et al, indicate that these protective effects might be attributable to reverse causality or the influence of confounders. Overall, our findings provide further support of alcohol as a critical mechanism in stroke. Furthermore, our findings support guidance to limit alcohol consumption for the prevention of stroke.

The current study has several strengths. As one of the largest targeted sequencing studies of ischemic stroke to date, power was enhanced to detect not only variant main effects but their interactions with important clinical and lifestyle risk factors. To our knowledge, this study also represents the only large‐scale sequencing study conducted in an East Asian ancestry population, allowing us to carefully assess ancestry specific variants. By examining homogeneous Han Chinese samples in the discovery and replication stages, population stratification was minimized. Furthermore, stringent quality control measures were employed for sequencing, genotyping, and covariable and outcome measurements. Still, certain limitations should also be mentioned. The sample size in the discovery stage was relatively small, limiting power to identify rare variants. Furthermore, the “extreme‐trait” design and more general “winner’s curse” may have resulted in somewhat overestimated effect sizes in the discovery‐stage analysis.^61^ Because discovery‐stage participants had more severe disease compared with replication‐stage participants, it is possible that heterogeneity in disease etiology was present across analytic stages, which could have led to a failure to replicate true signals. Furthermore, although frequency matching was successful in the discovery stage, regional imbalances were identified in the replication stage. However, these differences should have been accounted for when adjusting for province (within regions) in the statistical analysis. In addition, to maximize study efficiency, we only sequenced genes or genes in genomic regions previously related to atherosclerosis phenotypes. By design, this limited our ability to identify any novel stroke loci. Also, only exonic variants, according to the latest genome build, were selected for replication study. Among them, several were not successfully genotyped in the replication stage. Noncoding variants with important regulatory effects and coding variants that could not be successfully genotyped by the current study could functionally influence stroke susceptibility and warrant future follow‐up.^62^ Because environmental and clinical risk factors vary substantially by geography and associate with stroke, we employed geography matching and further adjusted for province in our analysis. This should minimize heterogeneity in environmental and clinical factors, particularly in the replication stage, where cases were selected from multiple studies conducted across various regions of China. However, similar to other observational studies, we cannot rule out residual confounding in our analyses.

In conclusion, this large‐scale targeted sequencing study implicated 3 loci—*FADS1‐FADS2*, *MTHFR*, and *ALDH2*—in ischemic stroke. By identifying variants at the *FADS1‐FADS2* locus, we provide the first genetic evidence for a causal, protective effect of PUFAs on ischemic stroke in an East Asian population. Furthermore, the observed deleterious effect of *MTHFR* variant rs180113 on stroke provides some of the first robust genomic evidence for a causal role of elevated homocysteine in this condition. We are also among the first to describe an interaction of the *ALDH2* rs671 variant and alcohol intake on ischemic stroke, bolstering support for the potentially harmful etiologic effects of excessive alcohol consumption on this condition. In total, our findings provide valuable mechanistic information on ischemic stroke susceptibility and highlight the synergistic effects of genetic and environmental factors in determining risk of ischemic stroke.

## Sources of Funding

This study was funded by National Natural Science Foundation of China (grant number: 81320108026) and a Project of the Priority Academic Program Development of Jiangsu Higher Education Institutions, China.

## Disclosures

None.

## Supporting information

Tables S1–S21
Figure S1–S2
