# Supplementary material for: Large‐Scale Targeted Sequencing Study of Ischemic Stroke in the Han Chinese Population
Source: J Am Heart Assoc. 2022 Oct 3;11(19):e025245. doi: 10.1161/JAHA.122.025245 (PMC9673712; doi:10.1161/JAHA.122.025245)

SUPPLEMENTAL MATERIAL

Table S1. Candidate genes studies or genome-wide association studies of ischemic stroke

Fist author	Journal	Publication year	Study method	Study design	Sample type	Study population	Sample Size	Main Results
Rubattu S	Circulation	1999	case-control	candidate gene	population based	European	796	rs5063
Endler G	Br J Haematol	2000	case-control	candidate gene	population based	European	176	rs1799768
Imai Y	Atherosclerosis	2000	case-control	candidate gene	population based	Asian	666	rs662
Kokubo Y	Stroke	2000	case-control	candidate gene	population based	Asian	1448	*ApoE* epsilon2
Reiner AP	Stroke	2000	case-control	candidate gene	population based	European	382	rs1126643
Baker RI	Blood	2001	case-control	candidate gene	population based	Austrilia	424	rs2243093
Bang Co	Cerebrovasc Dis	2001	case-control	candidate gene	population based	Asian	160	rs1799889
Dai K	Thromb Res	2001	case-control	candidate gene	population based	Asian	220	*vWF* Sma I polymorphism
Herrmann SM	Arterioscler Thromb Vasc Biol	2001	case-control	candidate gene	population based	European	560	rs45567233
Morrison AC	Stroke	2001	cohort	candidate gene	population based	European	1215	rs5443
Shen N	Zhonghua Yi Xue Za Zhi	2001	case-control	candidate gene	population based	Asian	219	HLA-DQA1*0301
Wu Y	Kobe J Med Sci	2001	case-control	candidate gene	population based	Asian	137	rs1801133
Morrison AC	Genet Epidemiol	2002	case-control	candidate gene	population based	European	1182	rs328
Revilla M	Neurosci Lett	2002	case-control	candidate gene	population based	European	164	rs1800795
Voetsch B	Stroke	2002	case-control	canddiate gene	population based	European	236	rs662
Chi LQ	Zhonghua Yi Xue Yi Chuan Xue Za Zhi	2003	case-control	candidate gene	population based	Asian	163	*GR* G1666T
Hoekstra T	Stroke	2003	cohort	candidate gene	population based	European	637	rs1799768
Kolovou GD	Angiology	2003	case-control	candidate gene	population based	European	405	ApoE epsilon2
Li Z	Stroke	2003	case-control	candidate gene	population based	Asian	3664	rs1801133
Martiskainen M	Stroke	2003	case-control	candidate gene	population based	European	132	rs1800790

Souza DR	Arg Neuropsiquiatr	2003	case-control	candidate gene	population based	European	414	rs429358 and rs7412
Wang XY	Zhonghua Yi Xue Yi Chuan Xue Za Zhi	2003	case-control	candidate gene	population based	Asian	162	rs7493
Xia J	Zhonghua Yi Xue Za Zhi(Taipai)	2003	case-control	candidate gene	population based	Asian	230	rs1801692
Zhao SP	Clin Chim Acta	2003	case-control	candidate gene	population based	Asian	213	rs328
Aznar J	Thromb Haemost	2004	case-control	candidate gene	population based	European	343	rs1799963
Baum L	Clin Chem Lab Med	2004	case-control	candidate gene	population based	Asian	545	rs1801133
Cipollone F	JAMA	2004	case-control	candidate gene	population based	European	1728	rs20417
Dong QL	Zhonghua Yi Xue Yi Chuan Xue Za Zhi	2004	case-control	candidate gene	population based	Asian	176	rs1800790
Frikke-Schmidt R	Eur Heart J	2004	cohort	candidate gene	population based	European	9238	rs11669576
Jannes J	Stroke	2004	case-control	candidate gene	population based	European	483	rs2020918
KöIsch H	Neurology	2004	case-control	candidate gene	population based	European	442	rs4925
Lanca V	Rev Port Cardiol	2004	case-control	candidate gene	population based	European	184	rs4646903
Lee BC	Neurosci Lett	2004	case-control	candidate gene	population based	Asian	317	rs18004629
Muñoz X	Hum Mutat	2004	case-control	candidate gene	population based	European	173	*GAS6* c.834+7G>A
Rubattu S	J Hypertens	2004	case-control	candidate gene	population based	European	451	rs1799752
Rubattu S	Stroke	2004	case-control	candidate gene	population based	European	442	rs5065
Santamaria A	Stroke	2004	case-control	candidate gene	population based	European	436	rs1801020
Slowik A	Stroke	2004	case-control	candidate gene	population based	European	276	rs5918
Suzuki Y	Neurology	2004	cross-sectional	candidate gene	population based	Asian	2195	ADH2*1 allele
Voetsch B	Arch Neurol	2004	case-control	canddiate gene	population based	European	236	rs705379
Wallerstedt SM	J Hypertens	2004	case-control	candidate gene	population based	European	1032	rs16139
Alluri RV	Eur J Neurol	2005	case-control	candidate gene	population based	India	118	rs1801133

Bevan S	Stroke	2005	case-control	candidate gene	population based	European	1013	rs40512, rs26950, rs702531, and rs829259
Champrro A	Cerebrovasc Dis	2005	case-control	candidate gene	population based	European	194	rs1800795
Chen J	J Mol Med(Berl)	2005	case-control	candidate gene	population based	Asian	1234	rs2794521
Dziedzic T	Cerebrovasc Dis	2005	case-control	candidate gene	population based	European	349	rs16944
Howard TD	Stroke	2005	case-control	candidate gene	population based	African	124	rs1800779 and rs2070744
Iacoviello L	Arterioscler Thromb Vasc Biol	2005	case-control	candidate gene	population based	Asian	268	rs16944
Lavergne E	Arterioscler Thromb Vasc Biol	2005	case-control	candidate gene	population based	European	929	rs3732378
Lin YC	Atherosclerosis	2005	case-control	candidate gene	population based	Asian	457	*TLR4* C119A
Lõhmussaar E	Stroke	2005	case-control	candidate gene	population based	European	1375	rs10507391
Ranade K	Stroke	2005	cohort	candidate gene	population based	European	2634	rs662
Rubattu S	Thromb Haemost	2005	case-control	candidate gene	population based	European	580	*F7* C122T
Saleheen D	Stroke	2005	case-control	candidate gene	population based	Asian	450	rs966221
Shearman AM	Stroke	2005	cohort	candidate gene	population based	European	2709	rs2234693
Slowik A	Cerebrovasc Dis	2005	case-control	candidate gene	population based	European	201	rs5985
Staton J	Stroke	2005	case-control	candidate gene	population based	European	315	rs3024718
van Rijn MJ	Neurology	2005	cross-sectional	candidate gene	family based	European	464	rs12188950 and rs3887175
Wiklund PG	Stroke	2005	case-control	candidate gene	population based	European	542	rs1799889
Zhang X	Zhonghua Yi Xue Yi Chuan Xue Za Zhi	2005	case-control	candidate gene	population based	Asian	102	*PAFAH1B1* 994C/T
Andrikovics H	Cerebrovasc Dis	2006	case-control	candidate gene	population based	European	394	rs2066718 and rs2230806
Baum L	Clin Chem Lab Med	2006	case-control	candidate gene	population based	Asian	816	rs328
Brophy VH	Stroke	2006	case-control	candidate gene	population based	European	485	5 SNPs of *PDE4D* gene
Funk M	Endler G	2006	case-control	candidate gene	population based	European	481	rs510317

Hegener HH	Clin Chem	2006	case-control	candidate gene	population based	European	518	rs266729 and rs182052
Hermans MP	Diabet Med	2006	cross-sectional	candidate gene	population based	European	165	rs1801133
Kaneko Y	Hypertens Res	2006	case-control	candidate gene	population based	Asian	559	rs768963
Kim Y	Neurosci Lett	2006	case-control	candidate gene	population based	Asian	227	rs2249358
Kim Y	Stroke	2006	case-control	candidate gene	population based	Asian	478	rs1800470
Lai J	Neurol India	2006	case-control	candidate gene	population based	Asian	112	rs16944
Lee BC	Neurosci Lett	2006	case-control	candidate gene	population based	Asian	272	rs1801282
Sie MP	Stroke	2006	cohort	candidate gene	population based	European	6996	rs1800470 and rs1800469
Staton JM	J Neurol Neurosurg Psychiatry	2006	case-control	candidate gene	population based	European	315	rs1396476, rs2910829 and rs966221
Szolnoki Z	J Mol Neurosci	2006	case-control	candidate gene	population based	European	580	rs5186
van Rijn MJ	J Neurol Neurosurg Psychiatry	2006	cohort	candidate gene	population based	European	6808	*IGF1* 192bp/-
van Rijn MJ	Stroke	2006	cohort	candidate gene	population based	European	6471	rs4961
Woo D	Stroke	2006	case-control	candidate gene	population based	Mixed	839	rs2910829 and rs152312
Yamada Y	Arterioscler Thromb Vasc Biol	2006	case-control	candidate gene	population based	Asian	2927	rs1800796
Yamaguchi S	Int J Mol Med	2006	case-control	candidate gene	population based	Asian	2705	rs235326, rs2107538 and rs4680
Zee RY	Stroke	2006	case-control	candidate gene	population based	European	518	rs702553
Zhang SY	Zhongguo Wei Zhong Bing Ji Jiu Yi Xue	2006	case-control	candidate gene	population based	Asian	61	*HLA-DRB1**0301
Zhang WL	Yi Chuan Xue Bao	2006	case-control	candidate gene	population based	Asian	1478	*ALOX5AP* SG13S114T/A
Zhu XY	Zhonghua Yi Xue Yi Chuan Xue Za Zhi	2006	case-control	candidate gene	population based	Asian	272	*LCAT* 608C/T
Abboud S	Plos one	2007	case-control	candidate gene	population based	European	563	rs505151
Alanne M	Hum Genet	2007	cohort	candidate gene	population based	European	14140	rs7178239
Benn M	J Clin Endocrinol Metab	2007	cohort	candidate gene	population based	European	9157	rs1042031

Berger K	Hum Genet	2007	case-control	candidate gene	population based	European	3648	rs1799864, rs1295686, rs1062535, rs1799983, and rs6131
Cole JW	BMC Neurol	2007	case-control	candidate gene	population based	European	194	rs6797312
Djoussé L	Am Heart J	2007	case-control	candidate gene	population based	European	1451	rs28362459
Fu Y	Zhonghua Yi Xue Za Zhi	2007	case-control	candidate gene	population based	Asian	245	rs1800588
Grewal RP	BMC Med Genet	2007	case-control	candidate gene	population based	African	879	*NOS3* repeat of a 27-bp
Hata J	Hum Mol Genet	2007	cohort	candidate gene	population based	Asian	2224	rs9943582
Kaushal R	Hum Genet	2007	case-control	candidate gene	population based	European	839	rs9579646 and rs4769874
Kuroda J	Eur J Neurol	2007	case-control	candidate gene	population based	Asian	1189	rs4673
Lee C	Stroke	2007	case-control	candidate gene	population based	Asian	782	rs16135
Liu J	Clin Chim Acta	2007	case-control	candidate gene	population based	Asian	232	rs1043618
Matarin M	Lancet Neurol	2007	case-control	GWAS	population based	European	517	27 loci with $P<1\times10-5$
Moon KS	J Mol Neurosci	2007	case-control	candidate gene	population based	Asian	729	T6235C *CYP1A1* polymorphism
Parfenov MG	J Neurol Sci	2007	case-control	candidate gene	population based	European	208	rs769446, rs42938, and rs7412
Quarta G	J Investig Med	2007	case-control	candidate gene	population based	European	451	rs708272
Saidi S	J Stroke Cerebrovasc Dis	2007	case-control	candidate gene	population based	African	253	rs1799768
Slowik A	Cerebrovasc Dis	2007	case-control	candidate gene	population based	European	276	rs7493
Stanzione R	Am J Hypertens	2007	case-control	candidate gene	population based	European	580	rs1042714
Tseng CH	Eur J Clin Invest	2007	case-control	candidate gene	population based	Asian	450	rs4646994
Voetsch B	Stroke	2007	case-control	canddiate gene	population based	European	246	*GPx-3* gene promoter haplotype
Volcik KA	Atherosclerosis	2007	cohort	canddiate gene	population based	African	3330	rs2228315
Worrall BB	Stroke	2007	case-control	candidate gene	population based	European	886	rs419598
Zhang Y	Clin Chim Acta	2007	case-control	candidate gene	population based	Asian	285	*GP* Iba VNTR polymorphism

Abboud S	Eur J Hum Genet	2008	case-control	candidate gene	population based	European	563	rs405509 and rs440446
Banerjee	Brain Res Bull	2008	case-control	candidate gene	population based	Asian	388	rs1800587 and rs966221
Can Demirdöğen B	Clin Biochem	2008	case-control	candidate gene	population based	European	186	rs662
Cheng J	Acta Neurol Scand	2008	case-control	candidate gene	population based	Asian	618	rs2229765
Cheng J	Clin Chim Acta	2008	case-control	candidate gene	population based	Asian	618	rs1800779 and rs2070744
Fatar M	Cerebrovasc Dis	2008	case-control	candidate gene	population based	European	340	rs1030868, rs2241145, rs2287074, rs2287076, and rs7201
Fava C	Hypertension	2008	cohort	candidate gene	population based	European	5753	rs2108622
Genius J	Cerebrovasc Dis	2008	case-control	candidate gene	population based	European	297	rs4673
Gretarsdottir S	Ann Neeurol	2008	case-control	GWAS	population based	European	36370	rs2200733
Gschwendtner A	Stroke	2008	case-control	candidate gene	population based	European	1337	rs751141, rs7357432, and rs2291635
Hagiwara N	Eur J Neurol	2008	case-control	candidate gene	population based	Asian	1492	rs2281939
Han SH	Blood Coagul Fibrinolysis	2008	case-control	candidate gene	population based	Asian	284	rs7950273
Jood K	J Thromb Haemost	2008	case-control	candidate gene	population based	European	1200	rs6050, rs2070011, rs2066864, rs1049636 and rs1800792
Kohsaka S	Atherosclerosis	2008	cohort	candidate gene	population based	African	3462	rs20417
Lazaros L	Acta Neurol Scand	2008	cross-sectional	candidate gene	population based	European	370	rs2234693 and rs9340799
Lin Y	BMC Med Genet	2008	case-control	candidate gene	population based	Asian	513	rs4903565
Maasz A	Circ J	2008	case-control	candidate gene	population based	Asian	509	rs662799
Maász A	J Neurol	2008	case-control	candidate gene	population based	European	295	rs3135506
Mararin M	Stroke	2008	case-control	candidate gene	population based	European	517	rs10116277 and rs1333042
Matarin M	J Cereb Blood Flow Metab	2008	case-control	candidate gene	population based	European	747	rs3756541 and rs2303124
Matarin M	Stroke	2008	case-control	candidate gene	population based	European	517	rs1333040 and rs2383207
Moe KT	Eur J Neurol	2008	case-control	candidate gene	population based	Asian	327	rs1801133

Möllsten A	J Hypertens	2008	case-control	candidate gene	population based	European	824	rs4646994 and rs5186
Morrison AC	Cerebrovasc Dis	2008	cohort	candidate gene	population based	African	3814	rs3213646, rs1042164, rs7439293, and rs11628722
Munshi A	J Neurol Sci	2008	case-control	candidate gene	population based	Asian	624	rs4646994
Naganuma T	Hereditas	2008	case-control	candidate gene	population based	Asian	227	rs699473
Pruissen DM	Blood	2008	case-control	candidate gene	population based	European	957	rs6003
Saidi S	Cerebrovasc Dis	2008	case-control	candidate gene	population based	Asian	634	rs1801106 and rs5918
Shi C	Clin Chim Acta	2008	case-control	candidate gene	population based	Asian	196	*eNOS* 4ab variant
Smith NL	J Thromb Haemost	2008	case-control	candidate gene	population based	European	3057	rs1800291 and rs1936645
Trompet S	Exp Gerontol	2008	cohort	candidate gene	population based	European	5389	rs1041981
Yamada Y	Int J Mol Med	2008	case-control	candidate gene	population based	Asian	1284	17 loci from 8 genes
Yamada Y	Stroke	2008	case-control	candidate gene	population based	Asian	2892	rs1800977, rs3027898, rs1059703, and
Zafarmand MH	Hypertension	2008	cohort	candidate gene	population based	European	15236	rs4961
Zhang K	Zhonghua Yi Xue Yi Chuan Xue Za Zhi	2008	case-control	candidate gene	population based	Asian	588	rs662799
Zhang L	Pharmacogenet Genomics	2008	case-control	candidate gene	population based	Asian	550	rs751141
Al-Allawi NA	Neurol India	2009	case-control	candidate gene	population based	Asian	120	rs1801133
Almawi WY	J Stroke Cerebrovasc Disease	2009	case-control	candidate gene	population based	Asian	238	rs1801133
Celiker G	Clin Appl Thromb Hemost	2009	case-control	candidate gene	population based	European	269	rs1799752
Chen J	Clin Sci(Lond)	2009	case-control	candidate gene	population based	Asian	2001	rs3739390
DE Gaetano M	J Thromb Haemost	2009	case-control	candidate gene	population based	European	815	rs1324214
Demirdöğen BC	Cell Biochem Funct	2009	case-control	candidate gene	population based	European	277	rs705379
Goracy I	J Appl Genet	2009	case-control	candidate gene	population based	European	285	rs1801133
Greisenegger S	Clin Chem	2009	case-control	candidate gene	population based	European	918	rs17611

Gschwendtner A	Ann Neurol	2009	case-control	candidate gene	population based	European	5226	rs7044859, rs496892, rs564398, rs7865618, rs1537378, rs2383207, and rs10757278
Gudbjartsson DF	Nat Genet	2009	case-control	candidate gene	population based	European	46133	rs7193343
Haidari M	Cerebrovasc Dis	2009	case-control	candidate gene	population based	European	712	*E-selectin* S128R and L554F polymorphisms
Hsieh FI	Diabetes Care	2009	case-control	candiate gene	population based	Asian	1074	*PPARgamma* C2821T
Hu WL	Brain Res Bull	2009	case-control	candiate gene	population based	Asian	536	rs3731245 and rs2383206
Ikram MA	N Engl J Med	2009	cohort	GWAS	population based	European	19602	rs11833579 and rs12425791
Karvanen J	Genet Epidemiol	2009	case-control	candidate loci	population based	European	33282	rs1333049 and rs11670734
Kim NS	Clin Biochem	2009	case-control	candidate gene	population based	Asian	504	*PON1* 1266G/A
Li XX	Mol Biol Rep	2009	case-control	candidate gene	population based	Asian	618	rs5498
Luke MM	Cerebrovasc Dis	2009	case-control	candidate gene	population based	European	1377	rs10757274, rs20455, rs3900940, and rs1010
Luke MM	Stroke	2009	case-control	candidate gene	population based	Mixed	5244	7 SNPs among White, 5 SNPs among Black
MacClellan LR	Stroke	2009	case-control	candidate gene	population based	European	327	rs10478723, rs1800542, rs10507875 and rs4885493
Munshi A	J Neurol Sci	2009	case-control	candidate gene	population based	Asian	500	rs966221
Naqanuma T	Clin Biochem	2009	case-control	candidate gene	population based	Asian	486	rs1760944, rs3136814, and rs1130409
Park SA	BMB Rep	2009	case-control	candidate gene	population based	Asian	713	rs12267682
Rezaii AA	Immunol Invest	2009	case-control	candidate gene	population based	Asian	309	rs380092
Saidi S	Acta Neurol Scand	2009	case-control	candidate gene	population based	African	773	rs699 and rs5051
Shimada T	Diabetes Res Clin Pract	2009	cross-sectional	candidate gene	population based	Asian	874	rs237025
Sieqerink B	J Thromb Haemost	2009	case-control	candidate gene	population based	European	957	rs6050
Smith JG	Circ Cardiovasc Genet	2009	case-control	candidate loci	population based	European	4565	rs10757274, rs2383207, and rs1333049
Sun JZ	Neurol India	2009	case-control	candidate gene	population based	Asian	215	rs1801133

Sun Y	Clin Sci(Lond)	2009	case-control	candidate gene	population based	Asian	1410	rs966221
Szolnoki Z	Clin Neurol Neurosurg	2009	case-control	candidate gene	population based	European	688	rs7291467 and rs909253
Um JY	J Mol Neurosci	2009	case-control	candidate gene	population based	Asian	801	rs2107538
Wahlstrand B	J Hypertens	2009	cohort	candidate gene	population based	European	5262	rs10757278
Wang B	J Neurol Sci	2009	case-control	candidate gene	population based	Asian	792	rs429358/rs7412
Wang Q	Acta Pharmacol Sin	2009	case-control	candidate gene	population based	Asian	1128	rs2794521
Wang XB	Zhongguo Shi Yan Xue Ye Xue Za Zhi	2009	case-control	candidate gene	population based	Asian	760	*PROCR* A6936G
Wang Z	Hypertens Res	2009	case-control	candidate gene	population based	Asian	2492	rs4944832
Wei YS	DNA Cell Biol	2009	case-control	candidate gene	population based	Asian	545	rs281865545
Wu L	Stroke	2009	case-control	candidate gene	population based	Asian	2383	rs2230500
Xu H	Clin Sci(Lond)	2009	case-control	candidate gene	population based	Asian	1140	rs966221
Yamada Y	Atherosclerosis	2009	case-control	GWAS	population based	Asian	6341	rs6007897, rs4044210, rs1671021, and rs1062708
Yamaguchi M	Med Sci Monit	2009	case-control	candidate gene	population based	Asian	466	rs670950
Zhang W	Stroke	2009	case-control	candidate gene	population based	Asian	3638	rs2305948 and rs2071559
Bai CH	J Biomed Sci	2010	case-control	candidate gene	family based	Asian	347	rs3074372
Buraczynska K	Clin Biochem	2010	case-control	candidate gene	population based	European	484	rs1024611
Chen J	Hum Mol Genet	2010	case-control	candidate gene	population based	Asian	1328	rs2507800
Chen K	Beijing Da Xue Xue Bao	2010	case-control	candidate gene	population based	Asian	1490	rs11833579
Deng S	Prog Neuropsychopharmacol Biol Psychiatry	2010	case-control	candidate gene	population based	Asian	652	rs2108622
Ding H	Circ Res	2010	case-control	candidate gene	population based	Asian	6885	*DDAH1* 4 bp -/+
Ding H	Pharmacogenet Genomics	2010	case-control	candidate gene	population based	Asian	2360	*CYP4A11* C296T and *CYP4F2* V433M

Ding H	Stroke	2010	case-control	candidate loci	population based	Asian	1680	rs10204475, rs10486776, and rs11052413
Domingues-Montanari S	Atherosclerosis	2010	case-control	candidate gene	population based	European	799	rs10947803
Domingues-Montanari S	Cerebrovasc Dis	2010	case-control	candidate gene	population based	European	1873	*ALOX5AP* SG13S114
Fava C	Pharmacogenet Genomics	2010	cohort	candidate gene	population based	European	5875	rs41507953
Freitas RN	Eur J Cardiovasc Prev Rehabil	2010	cohort	candidate gene	population based	European	20835	rs17238540
Giusti B	Thromb Haemost	2010	case-control	candidate gene	population based	European	1712	rs10037045, rs682985, rs1051319, rs202680, rs2274976, rs1979277, and rs20721958
Isordia-Salas I	Cerebrovasc Dis	2010	case-control	candidate gene	population based	Mexican	361	rs1801133
Kim NS	Clin Chim Acta	2010	case-control	candidate gene	population based	Asian	1350	rs16147
Kuhlenbaeumer G	Cerebrovasc Dis	2010	case-control	candidate gene	population based	European	3338	rs3093075, rs1130864, and rs1800947
Lai CQ	Am J Clin Nutr	2010	case-control	candidate gene	population based	European	1147	rs3851059 and rs7087728
Lee BC	Neurol Res	2010	case-control	candidate gene	population based	Asian	297	rs7535475 and rs7512140
Lee JD	Neurol Res	2010	case-control	candidate gene	population based	Asian	292	rs2738446 and rs2738450
Li N	Brain Res Bull	2010	case-control	candidate gene	population based	Asian	742	rs1800587 and rs966221
Li Y	Pharmacogenet Genomics	2010	case-control	candidate gene	population based	Asian	468	rs4646994
Lopaciuk S	Blood Coagul Fibrinolysis	2010	case-control	candidate gene	population based	European	300	rs6046
Majumdar V	Biochem Biophys Res Commun	2010	case-control	candidate gene	population based	Asian	1034	rs9536314
Majumdar V	J Atheroscler Thromb	2010	case-control	candidate gene	population based	Asian	126	*eNOS* intron 4a/b polymorphism
Matsushita T	Hum Mol Genet	2010	cohort	candidate gene	population based	Asian	2637	rs4376531 and rs2280887
Munshi A	Clin Chim Acta	2010	case-control	candidate gene	population based	Asian	780	rs2234693
Munshi A	Cytokine	2010	case-control	candidate gene	population based	Asian	950	rs1800896
Munshi A	J Neurol Sci	2010	case-control	candidate gene	population based	Asian	797	rs1799998

Nakazato T	J Hum Hypertens	2010	case-control	candidate gene	population based	Asian	405	rs3754701, rs3769048, and rs7590387
Saidi S	Acta Neurol Scand	2010	case-control	candidate gene	population based	African	773	rs1799983
Saidi S	J Renin Angiotensin Aldosterone Syst	2010	case-control	candidate gene	population based	Asian	773	rs1799998
Shyu HY	Clin Chim Acta	2010	case-control	candidate gene	population based	Asian	232	rs1800566
Shyu HY	Clin Chim Acta	2010	case-control	candidate gene	population based	Asian	232	*NQO1* C609T
Szilvási A	Genet Test Mol Biomarkers	2010	case-control	candidate gene	population based	European	154	rs4148211
Tong Y	Biochem Biophys Res Commun	2010	case-control	candidate gene	population based	Asian	1296	rs1800796
Tong Y	Clin Chim Acta	2010	case-control	candidate gene	population based	Asian	1496	rs1800629
Volcik KA	Stroke	2010	cohort	canddiate gene	population based	Mixed	12284	rs1799969
Xu C	Stroke	2010	case-control	candidate loci	population based	Asian	3984	rs11206510
Yamaguchi M	Hereditas	2010	case-control	candidate gene	population based	Asian	479	rs10780199
Yoshida T	Int J Mol Med	2010	case-control	candidate gene	population based	Asian	1884	rs9925481 and rs4923918
Yu JT	Clin Chim Acta	2010	case-control	candidate gene	population based	Asian	873	rs16147
Zhang N	Brain Res Bull	2010	case-control	candidate gene	population based	Asian	427	rs1946518
Bondarenko EA	Genetika	2011	case-control	candidate gene	population based	European	847	rs152312
Cheng YC	G3(Bethesda)	2011	case-control	GWAS	population based	European	1816	rs1986743 and rs2304556
Cheong MY	Yonsei Med J	2011	case-control	candidate gene	population based	Asian	1260	rs822391 and rs822396
Dahlberg J	J Hypertens	2011	case-control	candidate gene	population based	European	4505	rs1057293 and rs1743966
Fu Y	Eur Neurol	2011	case-control	candidate gene	population based	Asian	278	rs699947, rs1570360, and rs3025039
Gouveia LO	Atherosclerosis	2011	case-control	candidate gene	population based	European	1091	rs6007897 and rs4044210
Hata J	Nihon Eiseigaku Zasshi	2011	case-control	GWAS	population based	Asian	2224	rs9943582, rs4376531, and rs2230500
Hirose T	Hypertens Res	2011	cohort	candidate gene	population based	Asian	529	rs6609080

Ji R	Cerebrovasc Dis	2011	case-control	candidate gene	population based	Asian	1005	*ALOX5AP* -581_582 Ins A polymorphism
Kalita J	Clin Chim Acta	2011	case-control	candidate gene	population based	Asian	386	rs4646994
Kim JO	Stroke	2011	case-control	candidate gene	population based	Asian	1109	rs699947
Lee JD	J Clin Neurosci	2011	case-control	candidate gene	population based	Asian	569	rs4293222 and rs4360791
Leu HB	Atherosclerosis	2011	cohort	candidate gene	population based	Asian	3330	rs1764391
Li X	Eur J Intern Med	2011	case-control	candidate gene	population based	Asian	652	rs662799
Li XM	Nan Fang Yi Ke Da Xue Xue Bao	2011	case-control	candidate gene	population based	Asian	500	rs189897
Liu F	Mol Biol Rep	2011	case-control	candidate gene	population based	Asian	640	rs266729
Liu SY	Neurol India	2011	case-control	candidate gene	population based	Asian	293	rs660895
Luk AO	Atherosclerosis	2011	cohort	candidate gene	population based	Aisan	1327	rs2290608, rs1062535, rs328, and rs1799983
Meschia JF	Stroke	2011	case-control	GWAS	family based	European	332	No SNP achieved genome-wide significance, there was clustering of the most associated SNPs on chromosomes 3p (NOS1) and 6p.
Milton AG	Int J Stroke	2011	case-control	candidate gene	population based	European	481	rs152312
Munshi A	Eur J Neurol	2011	case-control	candidate gene	population based	Asian	1025	rs1800629
Oh SH	J Neurol Sci	2011	case-control	candidate gene	population based	Asian	979	rs1870377
Olsson S	Eur J Neurol	2011	case-control	candidate gene	population based	European	1512	rs2277984, rs3745565, and rs7857345
Peng Z	Lipids Health Dis	2011	case-control	candidate gene	population based	Asian	331	rs1800470 and rs1800469
Qin X	Clin Chim Acta	2011	case-control	candidate gene	population based	Asian	703	rs2107538
Sun H	Mol Biol Rep	2011	case-control	candidate gene	population based	Asian	1017	rs9579646
They-They TP	Acta Neurol Scand	2011	case-control	candidate gene	population based	European	224	rs1801133
Tong Y	Pharmacogenet Genomics	2011	case-control	canddiate loci	population based	Asian	1296	rs11833579
Tu Y	Pharmacogenet Genomics	2011	case-control	candidate gene	population based	Asian	1115	rs1799998

Wan XH	J Neurol Sci	2011	case-control	candidate gene	population based	Asian	1673	rs10849373
Wnuk M	Neurol Neurochir Pol	2011	case-control	candidate loci	population based	European	729	rs2200733
Yan JT	Acta Pharmacol Sin	2011	case-control	candidate gene	population based	Asian	115	rs841
Yin YY	Neurol India	2011	case-control	candidate gene	population based	Asian	597	rs4376531
Zakai NA	J Thromb Haemost	2011	cohort	candidate gene	population based	European	5255	rs3093261, rs6046, rs3781387, rs4918851, rs4648004, and rs3138055
Zhao D	J Mol Neurosci	2011	case-control	candidate gene	population based	Asian	703	rs805297 and rs805296
Arrequi M	Gene	2012	cohort	candidate loci	population based	European	1891	rs2943634
Babu MS	Gene	2012	case-control	candidate gene	population based	Asian	1029	rs4646972
Carty CL	Circ Cardiovasc Genet	2012	cohort	candidate loci	population based	Mixed	38816	5 SNPs were significant in AA; 2 SNPs were significant in EA; 1 SNP was significant in AI.
Chai Y	CAN Neurosci Ther	2012	case-control	candidate gene	population based	Asian	844	rs659366
Chen X	J Thromb Haemost	2012	case-control	candidate gene	population based	Asian	2528	rs4731702
Esen FI	Neurol Res	2012	case-control	candidate gene	population based	Turkish	451	rs2243093
Gui G	J Neuroinflammation	2012	case-control	candidate gene	population based	Asian	2415	rs1799964 and rs1800629
Harbuzova Viu	Fiziol Zh	2012	case-control	candidate gene	population based	European	294	rs1800801
He Y	Chin Med J(Engl)	2012	case-control	candidate gene	population based	Asian	800	rs918592
Holliday EG	Nat Genet	2012	case-control	GWAS	population based	European	56816	rs556621
ISGC Consortium	Nat Genet	2012	case-control	GWAS	population based	European	14033	rs11984041, rs2200733, and rs19065993
Jiang J	Vasc Med	2012	case-control	candidate gene	population based	Asian	427	rs10304
Jin X	J Int Med Res	2012	case-control	candidate gene	population based	Asian	801	rs2228314
Kim DE	BMC Res Notes	2012	case-control	candidate gene	population based	Asian	164	rs11833579
Kim JH	DNA Cell Biol	2012	case-control	candidate gene	population based	Asian	941	rs5067, rs5065, rs198372, and rs198373

Kim SK	J Interferon Cytokine Res	2012	case-control	candidate gene	population based	Asian	784	rs520540, rs602128, and rs679620
Liu Y	Clin Exp Hypertens	2012	case-control	candidate gene	population based	Asian	658	rs696831
Manso H	Atherosclerosis	2012	case-control	candidate gene	population based	European	1081	rs2293050, rs2139733, rs7308402, and rs1483757
Markoula S	J Neurol Sci	2012	case-control	candidate gene	population based	European	854	rs4986938
Munshi A	Gene	2012	case-control	candidate gene	population based	Asian	1029	rs12153798 and rs702553
Munshi A	J Neurol Sci	2012	case-control	candidate gene	population based	Asian	1025	rs320
Munshi A	Mol Biol Rep	2012	case-control	candidate gene	population based	Asian	994	rs2108622
Olsson S	Stroke	2012	case-control	candidate gene	population based	European	6450	rs380092
Pedersen A	Thromb Res	2012	case-control	candidate gene	population based	European	1512	rs8176592 and rs8176541
Polonikov A	Hypertens Res	2012	case-control	candidate gene	population based	European	667	rs713041
Polonikov A	J Mol Neurosci	2012	case-control	candidate gene	population based	European	667	*GSTT1* deletion
Shetova IM	Zh Nevrol Psikhiatr Im S S Korsakova	2012	case-control	GWAS	population based	European	950	rs1842993
Shyu HY	J Atheroscler Thromb	2012	case-control	candidate gene	population based	Asian	431	rs13181
Traylor M	Lancet Neurol	2012	case-control	GWAS	population based	European	74393	verified 2 previous accosiations at $P<5\times10^{-8}$, and identified 12 potentially novel loci at$P<5\times10^{-6}$
Yan G	Neurol India	2012	case-control	candidate gene	population based	Asian	582	rs1799998
Yuan M	J Clin Neurosci	2012	case-control	candidate gene	population based	Asian	504	rs2299939
Zhang HF	Mol Biol Rep	2012	case-control	candidate gene	population based	Asian	1435	rs1800449
Zhang W	Stroke	2012	case-control	candidate loci	population based	Asian	3321	rs10757278
Zhao DX	J Neurol Sci	2012	case-control	candidate gene	population based	Asian	703	rs5361
Zhao J	J Mol Neurosci	2012	case-control	candidate gene	population based	Asian	450	rs2619112 and rs7217186

Zhao N	J Neuroinflammation	2012	case-control	candidate gene	population based	Asian	2287	rs4795895 in overall; rs1799864 and rs1799987 in hypertensive group; rs3744508, rs730012, rs569108, rs1800469, and rs909253 in non-hypertensive group.
Ataman OV	Tsitol Genet	2013	case-control	candidate gene	population based	European	394	rs1800801
Biscetti F	Hum Genet	2013	case-control	candidate gene	population based	European	856	rs2073618, rs2073617, and rs3134069
Chehaibi K	J Mol Neurosci	2013	case-control	candidate gene	population based	Tunisian	388	rs2016520
Chen CC	Clin Biochem	2013	case-control	candidate gene	population based	Asian	1003	rs689466
Cui X	Cell Physiol Biochem	2013	case-control	candidate gene	population based	Asian	809	rs2070600
Dai Y	ScientificWorldJournal	2013	case-control	candidate gene	population based	Asian	890	rs7308402
Demirdöğen BC	Hum Exp Toxicol	2013	case-control	candidate gene	population based	Turkish	339	rs4646903
Feng J	J Thromb Thrombolysis	2013	case-control	candidate gene	population based	Asian	770	rs3861950
Guo L	BMC Med Genet	2013	case-control	candidate gene	population based	Asian	360	rs4742170, rs1929992, and rs10975519
Han Y	Exp Biol Med (Maywood)	2013	case-control	candidate gene	population based	Asian	1263	rs2389995 and rs2240419
Hanson E	Plos one	2013	case-control	candidate gene	population based	European	1512	rs3733403, rs925451, and rs1593
Heckman MG	Eur J Neurol	2013	case-control	candidate gene	population based	Mixed	1704	5 SNPs among Whites and 4 SNPs among Black
Jeon YJ	Arterioscler Thromb Vasc Biol	2013	case-control	candidate gene	population based	Asian	1121	rs29101642 and rs2292832
Jing M	Cell Physiol Biochem	2013	case-control	candidate gene	population based	Asian	928	rs11730582
Kawai T	J Atheroscler Thromb	2013	cohort	candidate gene	population based	Asian	353	rs1501299
Li R	J Mol Neurosci	2013	case-control	candidate gene	population based	Asian	718	*ECE-1b* C-338A
Li Y	CNS Neurosci Ther	2013	case-control	candidate gene	population based	Asian	646	rs653765
Liu H	J Clin Neurol	2013	case-control	candidate gene	population based	Asian	380	rs40593
Liu ME	Atherosclerosis	2013	case-control	candidate gene	population based	Asian	1344	rs3735590

Lu JX	Braz J Med Biol Res	2013	case-control	candidate gene	population based	Asian	750	rs1946518
Lu X	Hum Genet	2013	case-control	candidate gene	population based	Asian	2538	rs199469469
Ma AJ	Zhonghua Yi Xue Yi Chuan Xue Za Zhi	2013	case-control	candidate gene	population based	Asian	360	rs679620, rs522616, and rs3025058
Ma S	Biochem Genet	2013	case-control	candidate gene	population based	Asian	730	rs4918
Ma Y	Gene	2013	case-control	candidate gene	population based	Asian	622	*CD40* -1C/T
Oh SH	Clin Neurol Neurosurg	2013	case-control	candidate gene	population based	Asian	1128	rs28365031
Olsson S	Eur J Neurol	2013	case-control	candidate gene	population based	European	833	rs1035798
Park HJ	Int J Immunogenet	2013	case-control	candidate gene	population based	Asian	387	rs3117604
Ross OA	Plos one	2013	case-control	candidate gene	population based	European	1144	rs78501403, rs61749020, and rs3815188
Rubattu S	Eur J Intern Med	2013	case-control	candidate gene	population based	European	703	*NPR3* 55C>A
Shimizu M	J Clin Neurosci	2013	case-control	candidate gene	population based	Asian	264	rs4987262
Stepanyan A	Neurosci Lett	2013	case-control	candidate gene	population based	European	255	rs628117
Sun H	Chin Med J(Engl)	2013	case-control	candidate gene	population based	Asian	1423	rs2029253 and rs6538697
Tong YQ	Genet Mol Res	2013	case-control	canddiate gene	population based	Asian	200	*IL-4* VNTR polymorphism
Türkanoğlu Özçelik A	Gene	2013	case-control	candidate gene	population based	Turkish	390	rs2266782 and rs2266780
Williams FM	Ann Neurol	2013	case-control	candidate loci	population based	European	13167	rs505922
Xie G	Plos one	2013	case-control	candidate gene	population based	Asian	1475	rs1554286, rs3021094, and rs1800872
Zhang J	Genet Test Mol Biomarkers	2013	case-control	candidate gene	population based	Asian	1402	rs6050 and rs1800790
Zhang LJ	Lipids Health Dis	2013	case-control	candidate gene	population based	Asian	663	rs12218
Zhang Z	Biomed Res Int	2013	case-control	candidate gene	population based	Asian	926	rs1800587
Zhao J	Neurol Sci	2013	case-control	candidate gene	population based	Asian	677	rs768963
Zhong G	BMC Med Genet	2013	case-control	candidate gene	population based	Asian	836	rs705381 and rs854571

Zhu R	J Mol Neurosci	2013	case-control	candidate loci	population based	Asian	749	rs1746048 and rs501120
Bai Y	stroke	2014	case-control	candidate gene	population based	Asian	3471	rs2043211
Cai J	Zhejiang Da Xue Xue Bao Yi Xue Ban	2014	case-control	candidate gene	population based	Asian	608	rs11611246
Cao Y	Int J Neurosci	2014	case-control	candidate loci	population based	Asian	2492	*PAI-1* 4G/5G polymorphism
Chi LF	Neuroreport	2014	case-control	candidate gene	population based	Asian	551	rs776746
Choi HJ	Diabetes Res Clin Pract	2014	case-control	candidate gene	population based	Asian	810	rs7903146
Cotlarciuc I	Stroke	2014	case-control	candidate gene	population based	European	74393	rs9379800, rs17271121, rs12664474, rs2287921, rs1801131, and rs566295
Dichgans M	Stroke	2014	case-control	GWAS	population based	European	161388	21 SNPs with genome-wide significance
Gu S	BMC Med Genet	2014	case-control	candidate gene	population based	Asian	594	rs4986893 and rs4244285
Jiang B	Stroke	2014	case-control	candidate gene	population based	European	823	rs1799963
Li C	Zhonghua Yi Xue Yi Chuan Xue Za Zhi	2014	case-control	candidate gene	population based	Asian	1037	rs2236307
Li Y	Lipids Health Dis	2014	case-control	candidate gene	population based	Asian	409	rs35814191
Luo M	CNS Neurosci Ther	2014	case-control	candidate loci	population based	Aisan	1486	rs2208454
Nakamura K	Eur J Neurol	2014	case-control	candidate gene	population based	Asian	475	*GLA* 196G>C
Papapostolou A	Gene	2014	case-control	candidate gene	population based	European	423	rs4769055, rs3803277, and rs202068154
Wen D	Mol Neurobiol	2014	case-control	candidate loci	population based	Mixed	3548	*MMP-1* -1607 1G/2G and *MMP-3* -1612 5A/6A
Wu G	Int J Neurosci	2014	case-control	candidate loci	population based	Asian	4650	*ITGA2* C807T
Xiong X	Hum Genet	2014	case-control	candidate gene	population based	Asian	5792	rs1122608
Xu X	Zhonghua Yi Xue Yi Chuan Xue Za Zhi	2014	case-control	candidate gene	population based	Asian	586	rs1927911 and rs2149356
Yin C	J Clin Neurosci	2014	case-control	candidate gene	population based	Asian	567	rs351855
Zee RY	Clin Sci(Lond)	2014	cohort	candidate gene	population based	European	23294	10 SNPs from *ARHGEF10* and *ROCK1*

Zhao J	J Neurol Sci	2014	case-control	candidate loci	population based	Mixed	47026	*ACE* I/D polymorphism
Zhu R	Neurol Res	2014	case-control	candidate gene	population based	Asian	742	rs13290387
Zhu Y	J Thromb Thrombolysis	2014	case-control	candidate loci	family based	Asian	458	rs11833579
Au A	Sci Rep	2015	case-control	candidate loci	population based	Mixed	4016	rs11053646 and rs505151
Auer PL	JAMA Neurol	2015	case-control	exome sequencing	population based	Mixed	6000	*PDE4DIP* and ACOT4 genes
Bazina A	Gene	2015	case-control	candidate loci	population based	European	301	*IL-6* -174G>C polymorphisms
Bi J	J Stroke Cerebrovasc Dis	2015	case-control	candidate loci	population based	Asian	234	rs10757278, rs1537378, and rs1333047
Buraczynska K	Neuromolecular Med	2015	case-control	candidate loci	population based	Asian	732	*MMP-9* C(-1562)T
Carty CL	Stroke	2015	case-control	GWAS	population based	African	14746	rs4471613
Dang M	Neuromolecular Med	2015	case-control	candidate loci	population based	Asian	996	rs6438833
Fan Y	PLoS One	2015	case-control	candidate loci	population based	Asian	1835	rs17222919
Gao T	J Renin Angiotensin Aldosterone Syst	2015	case-control	candidate loci	population based	Asian	4290	*AGT* M235T and T174M
Hanscombe KB	Stroke	2015	case-control	GWAS	population based	Mixed	111913	*FXIIIB*
He P	Genet Mol Res	2015	case-control	candidate loci	population based	Asian	240	*APM-1* +276G/T
Li Q	J Stroke Cerebrovasc Dis	2015	case-control	candidate loci	population based	Asian	769	rs1378577 and rs57137919
Lv Q	Genet Mol Res	2015	case-control	candidate loci	population based	Asian	440	*MTHFR* A1298C
Oliveira-Filho J	J Stroke Cerebrovasc Dis	2015	case-control	candidate loci	population based	European	140	rs20417
Pereira NL	Circ Cardiovasc Genet	2015	case-control	candidate gene	population based	European	1784	rs5063
Shao J	Curr Neurovasc Res	2015	case-control	candidate loci	population based	Asian	373	rs768963
Su L	J Mol Neurosci	2015	case-control	candidate loci	population based	Asian	1632	rs2200733, and rs6843082
Tang H	J Stroke Cerebrovasc Dis	2015	case-control	candidate loci	population based	Asian	206	*LEPR* Lys109Arg and Gln223Arg
Yang Q	Int J Clin Exp Pathol	2015	case-control	candidate loci	population based	Asian	1669	rs174546 and rs174601

Yang Q	Lipids Health Dis	2015	case-control	candidate loci	population based	Asian	1065	rs2902940
Yi X	Gene	2015	case-control	candidate loci	population based	Asian	396	rs17110453 and rs751141
Yi X	J Stroke Cerebrovasc Dis	2015	case-control	candidate loci	population based	Asian	774	rs10507391 and rs776746
Yu Y	J Renin Angiotensin Aldosterone Syst	2015	case-control	candidate loci	population based	Mixed	5883	*CYP11B2* -344C/T
Zhang B	Genet Test Mol Biomarkers	2015	case-control	candidate loci	population based	Asian	774	rs9333025
Zhang Z	J Neurol Sci	2015	case-control	candidate loci	population based	Asian	846	rs12425791 and rs11833579
Zhang Z	Mol Neurobiol	2015	case-control	candidate loci	population based	Asian	919	*PRKCH* 1425G/A
Zhu XY	Int J Neurosci	2015	case-control	candidate loci	population based	Asian	4440	rs1801133
Cheng YC	Stroke	2016	case-control	GWAS	population based	Mixed	35221	rs11196288
He XW	J Neurol Sci	2016	case-control	candidate loci	population based	Asian	1540	rs4076317
Hinds DA	Hum Mol Genet	2016	case-control	GWAS	population based	European	74393	rs9797861
Kumar P	J Stroke Cerebrovasc Dis	2016	case-control	candidate loci	population based	Asian	500	*IL-10* -1082G/A
Lee TH	J Am Heart Assoc	2016	case-control	GWAS	population based	Asian	4292	rs2415317, rs934075, rs944289, rs2787417, and rs1952706
Malik R	Neurology	2016	case-control	GWAS	population based	European	29633	rs532436, rs2107595, rs2723334, and rs12932445
CHARGE Consortium, SiGN, and ISGC Consortium	Lancet Neurol	2016	case-control	GWAS	population based	Mixed	155765	rs12204590
SiGN; ISGC Consortium	Lancet Neurol	2016	case-control	GWAS	population based	Mixed	435001	rs12122341
Qiu S	PLoS One	2016	case-control	candidate loci	population based	Asian	5728	*VEGFR* +1192C>T and +1719A>T
Song Y	J Stroke Cerebrovasc Dis	2016	case-control	candidate loci	population based	Mixed	11270	*MTHFR* C677T
Sung YF	Stroke	2016	case-control	candidate loci	population based	Asian	1660	ALDH2*2
Traylor M	Neurology	2016	case-control	GWAS	population based	European	1976	genes from OXPHOS pathway
Williams SR	Neurology	2016	case-control	GWAS	population based	European	74393	rs3093068, rs16842599, and rs11265260

Zhang Z	Int J Neurosci	2016	case-control	candidate loci	population based	Asian	16672	rs12425791
Au A	Atherosclerosis	2017	case-control	candidate loci	population based	Mixed	8149	rs662799, rs3135506, rs1801701, rs1042031, and rs2230806
He T	J Stroke Cerebrovasc Dis	2017	case-control	candidate loci	population based	Mixed	6356	rs3918242
He T	J Stroke Cerebrovasc Dis	2017	case-control	candidate loci	population based	Mixed	4357	*LPL HindIII* variant
Lee TH	Sci Rep	2017	case-control	candidate loci	population based	Asian	2073	rs2594966, rs2594973, and rs4684776
Liu X	Genet Test Mol Biomarkers	2017	case-control	candidate loci	population based	Asian	772	*IL-10*-1082A/G
Malik R	Proc Natl Acad Sci U S A	2017	case-control	exome sequencing	population based	Mixed	12905	rs6647 and rs2023938
Rannikmäe K	Neurology	2017	case-control	candidate loci	population based	Mixed	57422	rs9515201 and rs79043147
Rodríguez-Esparragón F	Int J Neurosci	2017	case-control	candidate loci	population based	European	305	*PON2* S311C
Williams SR	Stroke	2017	case-control	GWAS	population based	European	2100	rs505922
Zhang G	J Stroke Cerebrovasc Dis	2017	case-control	candidate loci	population based	Mixed	6312	*MMP-1* -1607 1G/2G and *MMP-12* -82 A/G
Zhang L	Thromb Haemost	2017	case-control	candidate loci	population based	Asian	715	rs3024735 and 2273971
Zhang Z	Mol Neurobiol	2017	case-control	candidate loci	population based	Asian	914	rs2682818
Alhazzani AA	J Stroke Cerebrovasc Dis	2018	case-control	candidate loci	population based	Mixed	24885	*Factor V* G1691A
Bao MH	J Stroke Cerebrovasc Dis	2018	case-control	candidate loci	population based	Mixed	2319	rs6265
Cole JW	PLoS One	2018	case-control	candidate loci	population based	Mixed	26473	rs9574 and rs2069951
Li S	Curr Neurovasc Res	2018	case-control	candidate loci	population based	Mixed	2245	rs10435816, rs7025417, rs11792633, and rs7044343
Luo L	Biosci Rep	2018	case-control	candidate loci	population based	Asian	535	rs774320676 and rs928508030
Malik R	Ann Neurol	2018	case-control	GWAS	population based	Mixed	896016	rs1799983, rs9521634, and rs720470
Malik R	Nat Genet	2018	case-control	GWAS	population based	Mixed	521612	22 novel loci were identified
Misra S	Gene	2018	case-control	candidate loci	population based	Mixed	7389	*MMP-9* (-1562C/T) and *MMP-12* (-1082 A/G)
Mortensen JK	Cerebrovasc Dis	2018	case-control	candidate loci	population based	European	1405	rs25531

Nie F	Curr Neurovasc Res	2018	case-control	candidate loci	population based	Mixed	27203	rs12425791
Rao AS	Circ Genom Precis Med	2018	case-control	candidate loci	population based	European	337536	rs11591147
Khounphinith E	Int J Med Sci	2019	case-control	candidate loci	population based	Asian	1765	rs6882076
Luo H	J Stroke Cerebrovasc Dis	2019	case-control	candidate loci	population based	Mixed	11785	*FGβ* -148 C/T and -455 G/A
Ren Z	Aging (Albany NY)	2019	case-control	candidate loci	population based	Asian	4802	rs7703688
Wei YS	J Cell Mol Med	2019	case-control	candidate loci	population based	Asian	1127	rs2240183
Wu Y	BMC Med Genet	2019	case-control	candidate loci	population based	Asian	972	rs3093193, rs3093144 and rs12459936
Xiao T	Crit Rev Eukaryot Gene Expr	2019	case-control	candidate loci	population based	Asian	1080	rs12425791 and rs11833579
Zheng PF	BMC Cardiovasc Disord	2019	case-control	candidate loci	population based	Asian	1783	rs7819412
Zheng Z	Sci Rep	2019	case-control	candidate loci	population based	Asian	1157	rs12415607
Zuo S	Medicine (Baltimore)	2019	case-control	candidate loci	population based	Mixed	5127	*IL-10* -1082A/G
Han X	Cytokine	2020	case-control	candidate loci	population based	Asian	1157	rs10757278 and rs9333358
Jaworek T	Stroke	2020	case-control	exome sequencing	population based	Mixed	1449	*NAT10* gene
Keene KL	Stroke	2021	case-control	GWAS	population based	African	22000	24 loci were identified
Ken-Dror G	Ann Neurol	2021	case-control	GWAS	population based	European	2087	37 SNPs within the 9q34.2 region
Kumar A	Neurology	2021	case-control	GWAS	population based	Asian	5697	1p21, 16q24, 3p26 and 16p13
Liu C	BMC Cardiovasc Disord	2021	case-control	candidate loci	population based	Asian	547	rs4646188
Liu X	J Gene Med	2021	case-control	candidate loci	population based	Asian	610	rs966221
Traylor M	Lancet Neurol	2021	case-control	GWAS	population based	Mixed	262136	rs72934535, rs4621303, rs2293576, rs12445022, and rs9958650
Wang Q	Medicine (Baltimore)	2021	case-control	candidate loci	population based	Asian	1119	rs4977574
Yuan H	J Cardiovasc Pharmacol	2021	case-control	candidate loci	population based	Asian	977	rs12037987 and rs10776752
Hu Y	Stroke	2022	case-control	WGS	population based	Mixed	33949	7q22, *AUTS2*, 13q33, *RAP1GAP2*, and *TEX13C*

Table S2. Characteristics of participants according to region at discovery and replication stages.

Characteristics*	Discovery study			Replication study		
	Middle (n=589)	North (n=1411)	*P* value	Middle (n=5883)	North (n=4340)	*P* value
Age, years	65.9 ± 19.6	63.0 ± 8.2	<0.001	65.3 ± 9.9	61.5 ± 9.8	<0.0001
Male, n (%)	297 (50.4)	845 (49.9)	0.0001	3276 (55.7)	2137 (55.2)	0.6619
Smoking, n (%)	116 (19.7)	608 (43.1)	<0.001	2032 (34.6)	1338 (34.7)	0.9306
Drinking, n (%)	125 (21.2)	638 (45.2)	<0.001	1723 (29.7)	1027 (26.7)	0.0027
SBP, mm Hg	145.8 ± 24.5	148.0 ± 24.7	0.0726	140.7 ± 21.2	148.2 ± 22.3	<0.0001
DBP, mm Hg	85.0 ± 14.5	86.3 ± 13.8	0.0590	84.8 ± 11.3	87.3 ± 13.4	<0.0001
BMI, kg/m^2	24.0 ± 3.2	24.4 ± 3.5	0.0138	23.8 ± 3.4	24.8 ± 3.2	<0.0001
TC, mg/dL	195.5 ± 41.0	204.4 ± 43.6	<0.0001	184.8 ± 42.6	202.3 ± 44.4	<0.0001
TG, mg/dL	131.6 ± 75.6	147.7 ± 96.3	<0.0001	147.7 ± 124.5	168.5 ± 144.7	<0.0001
LDL, mg/dL	100.5 ± 33.3	120.2 ± 37.6	<0.0001	106.5 ± 32.4	123.1 ± 52.7	<0.0001
HDL, mg/dL	55.1 ± 14.9	52.6 ± 16.1	0.0009	50.3 ± 13.9	57.5 ± 44.4	<0.0001
FPG, mg/dL	108.3 ± 38.8	113.2 ± 42.3	0.0123	104.3 ± 38.7	113.8 ± 40.8	<0.0001
Obesity†, n (%)	51 (10.1)	197 (14.5)	0.0139	407 (10.2)	529 (14.6)	<0.0001
History of hypertension, n (%)	246 (41.8)	660 (46.8)	0.0403	2706 (46.2)	1912 (50.8)	<0.0001
History of hyperlipidemia, n (%)	27 (4.6)	57 (4.0)	0.6248	269 (4.9)	242 (11.1)	<0.0001
History of diabetes, n (%)	42 (7.1)	145 (10.3)	0.0284	1075 (18.3)	386 (10.1)	<000001

*Continuous variables are expressed as mean ± standard deviation. Categorical variables are expressed as number (percentage).
† Obesity was defined as BMI ≥ 28 kg/m^2

Table S3. Suggestive loci identified in the discovery stage main effects analyses (P <1×10^{-4})

**Variant**	**Chr**	**Position**	**Gene**	**CA/OA**	**CAF**	**Beta**	**SE**	***P* value**
rs1801133	1	11856378	*MTHFR*	A/G	0.55	0.37	0.07	1.54E-08
chr1_156647165	1	156647165	*NES*	CG/C	0.06	0.64	0.14	7.88E-06
chr2_102508323	2	102508323	*MAP4K4*	TG/T	0.08	0.68	0.13	1.01E-07
rs4512220	6	44923138	*SUPT3H*	C/G	0.28	0.28	0.07	8.90E-05
chr8_1818398	8	1818398	*ARHGEF10*	G/GA	0.06	0.67	0.15	7.26E-06
chr8_27403032	8	27403032	*EPHX2*	GC/G	0.04	1.20	0.20	7.71E-10
chr8_27403038	8	27403038	*EPHX2*	GC/G	0.06	0.60	0.14	2.31E-05
chr10_104718215	10	104718215	*CNNM2*	T/TG	0.04	0.85	0.19	8.80E-06
chr11_61537791	11	61537791	*MYRF*	GC/G	0.03	1.28	0.23	2.24E-08
rs60585534	14	65222652	*SPTB*	C/T	0.14	0.37	0.09	6.32E-05
chr14_100135024	14	100135024	*HHIPL1*	T/TG	0.02	1.08	0.27	4.72E-05
rs71116579	15	58885348	*LIPC; ADAM10*	A/ATTTTTTTTTTTTTTTTTTTTTTT	0.11	0.46	0.11	5.11E-05
chr16_73095267	16	73095267	*ZFHX3; HCCAT5*	T/TGCC	0.06	0.68	0.15	3.70E-06
chr16_73095268	16	73095268	*ZFHX3; HCCAT5*	G/GC	0.03	1.17	0.23	5.64E-07
chr17_46986377	17	46986377	*UBE2Z*	AC/A	0.03	0.85	0.21	6.07E-05
chr17_46986546	17	46986546	*UBE2Z*	TC/T	0.02	1.54	0.32	1.04E-06
chr19_51323750	19	51323750	*KLK1*	G/GAT	0.06	0.71	0.15	1.09E-06

CA=Coded allele; CAF= Coded allele frequency; Chr=chromosome; OA=Other allele

Table S4. Suggestive loci identified in the discovery stage 1 df interaction and 2df joint tests with sex (P <1×10^{-4})

Variant	Chr	Position	Gene	CA/OA	CAF	1 df interaction test			2df joint tests P-value
						Beta	SE	P-value	
rs1801133	1	11856378	*MTHFR*	A/G	0.55	0.12	0.13	3.69E-01	7.91E-08
chr1_156647165	1	156647165	*NES*	CG/C	0.06	-0.09	0.29	7.60E-01	4.60E-05
chr2_102508323	2	102508323	*MAP4K4*	TG/T	0.08	-0.50	0.26	5.71E-02	1.92E-07
chr8_1818398	8	1818398	*ARHGEF10*	G/GA	0.06	-0.18	0.30	5.63E-01	3.96E-05
chr8_27403032	8	27403032	*EPHX2*	GC/G	0.04	0.05	0.40	8.98E-01	5.86E-09
chr8_27403038	8	27403038	*EPHX2*	GC/G	0.06	0.33	0.29	2.57E-01	6.69E-05
chr10_104718215	10	104718215	*CNNM2*	T/TG	0.04	-0.23	0.40	5.68E-01	4.93E-05
chr11_61537791	11	61537791	*MYRF*	GC/G	0.03	0.24	0.46	6.00E-01	1.30E-07
chr14_100135024	14	100135024	*HHIPL1*	T/TG	0.02	0.94	0.55	9.01E-02	9.75E-05
chr16_73095267	16	73095267	*ZFHX3; HCCAT5*	T/TGCC	0.06	-0.14	0.30	6.33E-01	2.17E-05
chr16_73095268	16	73095268	*ZFHX3; HCCAT5*	G/GC	0.03	0.01	0.49	9.85E-01	3.65E-06
chr17_46986546	17	46986546	*UBE2Z*	TC/T	0.02	-0.15	0.64	8.09E-01	7.08E-06
chr19_51323750	19	51323750	*KLK1*	G/GAT	0.06	-0.51	0.29	8.37E-02	2.53E-06

Table S5. Suggestive loci identified in the discovery stage 1 df interaction and 2df joint tests with body-mass index (P <1×10^{-4})

Variant	Chr	Position	Gene	CA/OA	CAF	1 df interaction test			2df joint tests P-value
						Beta	SE	P-value	
rs1801133	1	11856378	*MTHFR*	A/G	0.55	-0.004	0.02	8.43E-01	3.51E-07
chr1_156647165	1	156647165	*NES*	CG/C	0.06	-0.04	0.05	3.82E-01	3.95E-05
rs4512220	6	44923138	*SUPT3H*	C/G	0.28	-0.02	0.02	5.04E-01	3.15E-05
chr8_1818398	8	1818398	*ARHGEF10*	G/GA	0.06	0.08	0.06	1.24E-01	2.45E-05
chr8_27403032	8	27403032	*EPHX2*	GC/G	0.04	-0.04	0.06	5.70E-01	2.51E-06
chr11_61537791	11	61537791	*MYRF*	GC/G	0.03	-0.08	0.08	2.91E-01	1.67E-05
chr16_73095268	16	73095268	*ZFHX3; HCCAT5*	G/GC	0.03	-0.10	0.07	1.77E-01	2.87E-05

CA=Coded allele; CAF= Coded allele frequency; Chr=chromosome; OA=Other allele

Table S6. Suggestive loci identified in the discovery stage 1 df interaction and 2df joint tests with fasting plasma glucose (P <1×10^{-4})

Variant	Chr	Position	Gene	CA/OA	CAF	1 df interaction test			*2 df joint test P-value*
						Beta	SE	*P-value*	
rs1801133	1	11856378	*MTHFR*	A/G	0.55	-0.05	0.05	3.23E-01	2.79E-07
rs386600283	1	94998750	*F3*	A/G	0.02	-0.42	0.10	5.96E-05	1.09E-05
chr1_156647165	1	156647165	*NES*	CG/C	0.06	-0.15	0.09	9.96E-02	2.84E-05
chr2_102508323	2	102508323	*MAP4K4*	TG/T	0.08	0.04	0.10	6.96E-01	2.96E-06
rs146404695	5	58571684	*PDE4D*	C/T	0.02	-0.37	0.09	1.61E-05	1.89E-05
rs2071303	6	26091336	*HFE*	T/C	0.35	-0.15	0.05	1.69E-03	5.52E-05
rs144146728	6	49403301	*MUT*	T/C	0.03	-0.37	0.09	5.00E-05	1.99E-04
chr8_27403032	8	27403032	*EPHX2*	GC/G	0.04	0.00	0.16	9.99E-01	4.21E-08
rs7075480	10	100176627	*HPS1*	A/G	0.06	-0.31	0.08	6.89E-05	3.40E-04
rs12571249	10	100186959	*HPS1*	G/A	0.06	-0.31	0.08	9.07E-05	4.04E-04
chr11_61537791	11	61537791	*MYRF*	GC/G	0.03	0.11	0.20	5.95E-01	4.15E-07
rs11174397	12	62645916	*FAM19A2; USP15*	G/C	0.14	-0.28	0.06	1.04E-05	1.02E-05
rs11174398	12	62646348	*FAM19A2; USP15*	C/T	0.14	-0.28	0.06	9.92E-06	1.03E-05
rs11174399	12	62646556	*FAM19A2; USP15*	A/G	0.14	-0.28	0.06	1.04E-05	1.02E-05
rs117545816	12	112148254	*ACAD10*	A/G	0.05	-0.33	0.09	9.98E-05	3.80E-04
rs4646779	12	112237981	*ALDH2*	C/T	0.03	-0.37	0.09	1.78E-05	9.98E-05
chr16_73095268	16	73095268	*ZFHX3; HCCAT5*	G/GC	0.03	0.09	0.22	6.95E-01	4.37E-06
rs2250526	17	57951973	*TUBD1*	A/G	0.42	-0.20	0.05	4.29E-05	4.83E-05
chr19_51323750	19	51323750	*KLK1*	G/GAT	0.06	-0.14	0.09	1.44E-01	7.50E-06
rs41345851	20	23063779	*CD93*	T/C	0.15	-0.23	0.05	1.38E-05	7.82E-05
rs41418351	20	23064912	*CD93*	C/T	0.15	-0.21	0.05	8.44E-05	4.39E-04

CA=Coded allele; CAF= Coded allele frequency; Chr=chromosome; OA=Other allele

Table S7. Suggestive loci identified in the discovery stage 1 df interaction and 2df joint tests with HDL-cholesterol (P <1×10^{-4})

Variant	Chr	Position	Gene	CA/OA	CAF	1 df interaction test			*2 df joint test*
						Beta	SE	*P-value*	*P-value*
rs1801133	1	11856378	*MTHFR*	A/G	0.55	-0.24	0.19	1.90E-01	1.64E-07
chr1_156647165	1	156647165	*NES*	CG/C	0.06	-0.06	0.36	8.78E-01	5.46E-05
rs3213768	2	53943696	*ASB3; GPR75-ASB3*	T/C	0.25	0.70	0.18	7.55E-05	1.07E-04
rs2287339	2	53992593	*ASB3; GPR75-ASB3*	T/A	0.25	0.71	0.18	5.19E-05	7.50E-05
rs76943041	2	53993382	*ASB3; GPR75-ASB3*	G/A	0.25	0.71	0.18	5.78E-05	6.52E-05
rs3755116	2	53993503	*ASB3; GPR75-ASB3*	A/G	0.25	0.70	0.18	7.28E-05	8.24E-05
rs3755115	2	53994160	*ASB3; GPR75-ASB3*	A/C	0.25	0.72	0.18	4.80E-05	6.03E-05
rs3755114	2	53994493	*ASB3; GPR75-ASB3*	G/A	0.25	0.72	0.18	4.22E-05	8.17E-05
rs76328173	2	54087470	*GPR75; GPR75-ASB3*	A/G	0.25	0.73	0.18	4.40E-05	3.86E-05
rs3755113	2	54088024	*GPR75*	T/C	0.25	0.69	0.18	1.03E-04	9.18E-05
chr2_102508323	2	102508323	*MAP4K4*	TG/T	0.08	0.67	0.28	1.81E-02	1.56E-07
rs76224543	2	240061403	*HDAC4*	T/C	0.04	1.43	0.34	2.39E-05	5.30E-05
rs4687994	3	119013558	*ARHGAP31*	A/G	0.33	0.84	0.18	3.33E-06	1.62E-05
rs1194182	7	80231504	*CD36*	G/C	0.37	-0.86	0.19	4.66E-06	2.70E-05
rs2366855	7	80253455	*CD36*	A/T	0.38	-0.84	0.19	9.96E-06	5.74E-05
rs73167652	7	150700637	*NOS3*	G/A	0.08	1.24	0.27	3.45E-06	9.39E-06
rs3730305	7	150704400	*NOS3*	A/C	0.08	1.22	0.27	4.24E-06	1.38E-05
chr8_1818398	8	1818398	*ARHGEF10*	G/GA	0.06	-0.61	0.42	1.44E-01	5.30E-05
rs2916715	8	6357307	*ANGPT2*	C/T	0.10	0.99	0.25	7.67E-05	1.26E-05
rs1961222	8	6377433	*ANGPT2*	T/C	0.08	1.00	0.26	1.01E-04	3.76E-05
rs328	8	19819724	*LPL*	G/C	0.08	1.12	0.28	5.98E-05	4.68E-05

rs11570891	8	19822810	*LPL*	T/C	0.08	1.11	0.28	6.17E-05	6.51E-05
rs1803924	8	19823674	*LPL*	T/C	0.08	1.09	0.28	8.30E-05	6.88E-05
rs3735964	8	19824045	*LPL*	A/C	0.08	1.09	0.28	8.06E-05	6.52E-05
rs1059611	8	19824563	*LPL*	C/T	0.08	1.10	0.28	7.13E-05	6.11E-05
rs10645926	8	19824626	*LPL*	CTT/C	0.08	1.10	0.28	7.05E-05	5.59E-05
rs15285	8	19824667	*LPL*	T/C	0.08	1.07	0.28	1.50E-04	5.36E-05
chr8_27403032	8	27403032	*EPHX2*	GC/G	0.04	-0.07	0.51	8.85E-01	3.84E-08
chr10_104718215	10	104718215	*CNNM2*	T/TG	0.04	0.18	0.43	6.71E-01	9.91E-05
rs78414630	10	104842172	*CNNM2; NT5C2*	T/C	0.08	1.07	0.27	6.43E-05	3.33E-04
rs12573199	10	104848844	*NT5C2*	T/A	0.08	1.04	0.27	9.53E-05	4.93E-04
rs12573200	10	104848855	*NT5C2*	T/A	0.08	1.04	0.27	9.54E-05	4.89E-04
rs12573221	10	104849144	*NT5C2*	C/A	0.08	1.07	0.27	6.41E-05	3.39E-04
rs11191554	10	104855278	*NT5C2*	T/C	0.17	0.90	0.23	6.85E-05	1.21E-04
rs78436955	10	104900841	*NT5C2*	T/C	0.07	1.05	0.27	8.99E-05	4.65E-04
chr11_61537791	11	61537791	*MYRF*	GC/G	0.03	0.78	0.53	1.41E-01	3.65E-08
rs174528	11	61543499	*MYRF*	C/T	0.35	-0.53	0.20	9.85E-03	8.88E-06
rs174530	11	61546592	*MYRF*	G/A	0.34	-0.54	0.20	7.72E-03	2.86E-06
rs174533	11	61549025	*MYRF*	A/G	0.34	-0.53	0.21	1.01E-02	8.96E-06
rs174534	11	61549458	*MYRF*	G/A	0.34	-0.48	0.20	1.73E-02	1.44E-05
rs174535	11	61551356	*MYRF*	C/T	0.34	-0.53	0.20	1.00E-02	4.37E-06
rs174536	11	61551927	*MYRF*	C/A	0.34	-0.50	0.21	1.50E-02	8.94E-06
rs174537	11	61552680	*MYRF*	T/G	0.34	-0.51	0.21	1.28E-02	5.85E-06
rs174545	11	61569306	*FADS1*	G/C	0.33	-0.51	0.21	1.44E-02	9.95E-06
rs174546	11	61569830	*FADS1*	T/C	0.33	-0.53	0.21	9.99E-03	1.02E-05

rs174548	11	61571348	*FADS1*	G/C	0.33	-0.49	0.21	1.84E-02	1.01E-05
rs174549	11	61571382	*FADS1*	A/G	0.33	-0.47	0.21	2.33E-02	1.69E-05
rs174550	11	61571478	*FADS1*	C/T	0.33	-0.50	0.21	1.52E-02	7.72E-06
rs174560	11	61581764	*FADS1*	C/T	0.33	-0.47	0.21	2.46E-02	1.75E-05
rs174561	11	61582708	*MIR1908*	C/T	0.33	-0.46	0.21	2.59E-02	2.20E-05
rs174568	11	61593816	*FADS2*	T/C	0.33	-0.50	0.21	1.48E-02	1.85E-05
rs3834458	11	61594920	*FADS2*	C/CT	0.33	-0.51	0.21	1.38E-02	1.25E-05
rs174600	11	61622227	*FADS2*	C/T	0.35	-0.49	0.20	1.54E-02	1.86E-05
rs174601	11	61623140	*FADS2*	T/C	0.35	-0.43	0.20	3.56E-02	4.25E-05
rs97384	11	61624181	*FADS2*	T/C	0.35	-0.48	0.20	1.58E-02	6.67E-05
rs60585534	14	65222652	*SPTB*	C/T	0.14	0.48	0.20	1.45E-02	2.61E-05
rs3751542	15	58856033	*LIPC*	T/C	0.45	0.68	0.17	5.05E-05	2.43E-04
rs3829460	15	58857945	*LIPC*	T/A	0.45	0.72	0.17	1.73E-05	9.49E-05
chr16_73095267	16	73095267	*ZFHX3; HCCAT5*	T/TGCC	0.06	-0.78	0.47	9.66E-02	3.48E-05
chr16_73095268	16	73095268	*ZFHX3; HCCAT5*	G/GC	0.03	-0.20	0.58	7.33E-01	1.68E-05
rs1061228	16	88782079	*PIEZO1*	A/G	0.15	0.76	0.20	9.51E-05	2.02E-04
chr17_7577678	17	7577678	*TP53*	CT/C	0.05	0.88	0.28	1.37E-03	3.80E-06
chr17_46986546	17	46986546	*UBE2Z*	TC/T	0.02	0.66	0.70	3.44E-01	6.57E-06
rs6105	18	61565062	*SERPINB2*	G/C	0.07	1.11	0.27	2.77E-05	6.94E-05
chr19_15997151	19	15997151	*CYP4F2*	C/CA	0.02	0.95	0.48	4.65E-02	8.31E-05
rs838136	19	49256388	*FUT1*	C/T	0.37	-0.84	0.19	7.69E-06	2.17E-05
chr19_51323750	19	51323750	*KLK1*	G/GAT	0.06	0.08	0.38	8.37E-01	7.29E-06
chr19_51323764	19	51323764	*KLK1*	G/GCAT	0.04	0.94	0.33	5.07E-03	3.93E-05

CA=Coded allele; CAF= Coded allele frequency; Chr=chromosome; OA=Other allele

Table S8. Suggestive loci identified in the discovery stage 1 df interaction and 2df joint tests with LDL-cholesterol (P <1×10^{-4})

**Variant**	**Chr**	**Position**	**Gene**	**CA/OA**	**CAF**	**1 df interaction test**			***2 df joint test P-value***
						**Beta**	**SE**	***P-value***	
rs1801133	1	11856378	*MTHFR*	A/G	0.55	0.01	0.07	8.85E-01	5.17E-07
chr1_156647165	1	156647165	*NES*	CG/C	0.06	-0.06	0.15	6.66E-01	5.35E-05
chr2_102508323	2	102508323	*MAP4K4*	TG/T	0.08	0.21	0.13	1.04E-01	3.00E-06
rs9861471	3	79816891	*ROBO1*	C/G	0.37	-0.25	0.07	6.10E-04	6.99E-05
rs4512220	6	44923138	*SUPT3H*	C/G	0.28	0.16	0.08	4.21E-02	7.92E-05
chr8_1818398	8	1818398	*ARHGEF10*	G/GA	0.06	0.20	0.16	2.33E-01	1.20E-05
chr8_27403032	8	27403032	*EPHX2*	GC/G	0.04	0.33	0.21	1.18E-01	2.92E-08
chr8_27403038	8	27403038	*EPHX2*	GC/G	0.06	0.35	0.16	2.79E-02	3.80E-05
chr11_61537791	11	61537791	*MYRF*	GC/G	0.03	0.18	0.24	4.67E-01	1.75E-07
chr14_86098827	14	86098827	*FLRT2; LINC02328*	A/AT	0.04	0.78	0.22	4.52E-04	6.49E-05
chr16_73095268	16	73095268	*ZFHX3; HCCAT5*	G/GC	0.03	0.04	0.27	8.93E-01	5.63E-06
chr17_46986546	17	46986546	*UBE2Z*	TC/T	0.02	-0.28	0.36	4.44E-01	1.44E-05
chr19_51323750	19	51323750	*KLK1*	G/GAT	0.06	0.05	0.16	7.51E-01	3.13E-05
chr22_19954915	22	19954915	*COMT*	C/CA	0.02	1.72	0.40	2.19E-05	1.22E-04

CA=Coded allele; CAF= Coded allele frequency; Chr=chromosome; OA=Other allele

Table S9. Suggestive loci identified in the discovery stage 1 df interaction and 2df joint tests with triglycerides (P <1×10^{-4})

Variant	Chr	Position	Gene	CA/OA	CAF	1 df interaction test			***2 df joint test P-value***
						**Beta**	**SE**	***P-value***	
rs1801133	1	11856378	*MTHFR*	A/G	0.55	0.11	0.13	4.28E-01	1.98E-07
chr1_156647165	1	156647165	*NES*	CG/C	0.06	0.21	0.28	4.62E-01	6.88E-05
chr2_102508323	2	102508323	*MAP4K4*	TG/T	0.08	-0.03	0.26	9.10E-01	3.77E-06
chr8_1818398	8	1818398	*ARHGEF10*	G/GA	0.06	0.11	0.31	7.13E-01	9.88E-05
chr8_27403032	8	27403032	*EPHX2*	GC/G	0.04	-0.07	0.43	8.63E-01	1.24E-08
chr10_104718215	10	104718215	*CNNM2*	T/TG	0.04	0.76	0.44	8.61E-02	5.98E-05
chr11_61537791	11	61537791	*MYRF*	GC/G	0.03	0.58	0.50	2.47E-01	1.41E-07
rs174530	11	61546592	*MYRF*	G/A	0.34	-0.04	0.14	7.52E-01	7.63E-05
rs174535	11	61551356	*MYRF*	C/T	0.34	-0.05	0.14	7.25E-01	8.15E-05
rs174537	11	61552680	*MYRF*	T/G	0.34	-0.04	0.14	7.60E-01	9.30E-05
rs174550	11	61571478	*FADS1*	C/T	0.33	-0.06	0.14	6.66E-01	9.35E-05
chr16_73095267	16	73095267	*ZFHX3; HCCAT5*	T/TGCC	0.06	0.96	0.32	2.91E-03	3.14E-06
chr16_73095268	16	73095268	*ZFHX3; HCCAT5*	G/GC	0.03	-0.14	0.47	7.65E-01	5.24E-06
chr17_46986546	17	46986546	*UBE2Z*	TC/T	0.02	-0.28	0.66	6.71E-01	5.71E-06
chr19_51323750	19	51323750	*KLK1*	G/GAT	0.06	-0.31	0.30	3.02E-01	1.57E-06

CA=Coded allele; CAF= Coded allele frequency; Chr=chromosome; OA=Other allele

Table S10. Suggestive loci identified in the discovery stage 1 df interaction and 2df joint tests with alcohol drinking (P <1×10⁻⁴)

Variant	Chr	Position	Gene	CA/OA	CAF	1 df interaction test			*2 df joint test P-value*
						Beta	SE	*P-value*	
rs1801133	1	11856378	*MTHFR*	A/G	0.55	-0.33	0.14	1.69E-02	6.22E-09
chr1_156647165	1	156647165	*NES*	CG/C	0.06	0.01	0.30	9.84E-01	2.34E-05
chr1_182077613	1	182077613	*ZNF648; LINC01344*	CT/C	0.07	-1.27	0.31	5.52E-05	2.67E-04
rs4148217	2	44099433	*ABCG8*	A/C	0.12	0.85	0.21	6.67E-05	1.61E-04
chr2_102508323	2	102508323	*MAP4K4*	TG/T	0.08	0.60	0.28	3.00E-02	2.36E-06
chr8_1818398	8	1818398	*ARHGEF10*	G/GA	0.06	-0.38	0.32	2.34E-01	1.03E-05
chr8_27403032	8	27403032	*EPHX2*	GC/G	0.04	0.24	0.42	5.60E-01	1.82E-07
chr11_61537791	11	61537791	*MYRF*	GC/G	0.03	-0.16	0.50	7.56E-01	5.52E-06
rs3736211	12	24985637	*BCAT1*	C/T	0.12	0.83	0.21	8.36E-05	4.00E-04
rs60104197	12	24986679	*BCAT1*	A/G	0.12	0.85	0.21	4.84E-05	2.03E-04
rs12228257	12	24987129	*BCAT1*	A/G	0.12	0.83	0.21	7.37E-05	3.22E-04
rs3782886	12	112110489	*BRAP*	C/T	0.16	-0.88	0.23	1.19E-04	6.66E-05
rs4646776	12	112230019	*ALDH2*	C/G	0.16	-0.89	0.23	1.23E-04	8.51E-05
rs671	12	112241766	*ALDH2*	A/G	0.16	-0.95	0.23	5.47E-05	3.06E-05
rs78069066	12	112337924	*ADAM1A*	A/G	0.16	-0.83	0.23	2.45E-04	9.97E-05
chr16_73095267	16	73095267	*ZFHX3; HCCAT5*	T/TGCC	0.06	0.12	0.31	7.05E-01	4.41E-05
chr16_73095268	16	73095268	*ZFHX3; HCCAT5*	G/GC	0.03	-0.71	0.51	1.61E-01	1.63E-06
rs2664593	17	4545132	*ALOX15*	G/C	0.19	0.69	0.18	9.30E-05	2.86E-04
chr17_46986546	17	46986546	*UBE2Z*	TC/T	0.02	-0.36	0.68	5.96E-01	9.09E-05
chr19_51323750	19	51323750	*KLK1*	G/GAT	0.06	0.14	0.30	6.41E-01	3.94E-06
chr20_33762783	20	33762783	*PROCR*	G/GTCT	0.11	-0.52	0.24	2.99E-02	4.98E-05

rs8094	23	15415583	*PIR*	T/C	0.49	-0.92	0.17	8.12E-08	1.18E-07
rs6632666	23	15496733	*PIR-FIGF*	T/C	0.48	-0.87	0.17	3.49E-07	2.17E-06
rs2285666	23	15610348	*ACE2*	C/T	0.46	-0.68	0.17	5.75E-05	2.62E-04

CA=Coded allele; CAF= Coded allele frequency; Chr=chromosome; OA=Other allele

Table S11. Suggestive loci identified in the discovery stage 1 df interaction and 2df joint tests with cigarette smoking (P <1×10^{-4})

Variant	Chr	Position	Gene	CA/OA	CAF	1 df interaction test			*2 df joint test P-value*
						Beta	SE	*P-value*	
rs1801133	1	11856378	*MTHFR*	A/G	0.55	-0.22	0.14	1.12E-01	3.18E-08
chr1_156647165	1	156647165	*NES*	CG/C	0.06	-0.12	0.29	6.95E-01	3.86E-05
chr2_102508323	2	102508323	*MAP4K4*	TG/T	0.08	0.23	0.28	3.94E-01	5.82E-07
chr4_2930427	4	2930427	*ADD1*	GC/G	0.08	1.01	0.30	8.73E-04	6.25E-06
chr8_1818398	8	1818398	*ARHGEF10*	G/GA	0.06	-0.12	0.30	7.00E-01	3.41E-05
chr8_27403032	8	27403032	*EPHX2*	GC/G	0.04	1.03	0.48	3.03E-02	6.74E-09
chr8_27403038	8	27403038	*EPHX2*	GC/G	0.06	0.28	0.30	3.39E-01	9.38E-05
chr10_104718215	10	104718215	*CNNM2*	T/TG	0.04	-0.06	0.38	8.76E-01	4.41E-05
chr11_61537791	11	61537791	*MYRF*	GC/G	0.03	-0.24	0.49	6.30E-01	1.65E-07
rs60585534	14	65222652	*SPTB*	C/T	0.14	-0.33	0.19	8.14E-02	8.08E-05
chr16_73095267	16	73095267	*ZFHX3; HCCAT5*	T/TGCC	0.06	0.21	0.31	5.01E-01	1.84E-05
chr16_73095268	16	73095268	*ZFHX3; HCCAT5*	G/GC	0.03	-0.18	0.47	7.03E-01	3.39E-06
chr17_46986546	17	46986546	*UBE2Z*	TC/T	0.02	0.70	0.83	4.02E-01	9.81E-06
chr19_51323750	19	51323750	*KLK1*	G/GAT	0.06	0.47	0.31	1.25E-01	2.74E-06
rs8094	23	15415583	*PIR*	T/C	0.49	-0.77	0.16	1.28E-06	1.96E-06
rs6632666	23	15496733	*PIR-FIGF*	T/C	0.48	-0.70	0.16	1.18E-05	6.42E-05
rs2285666	23	15610348	*ACE2*	C/T	0.46	-0.63	0.16	9.01E-05	3.73E-04
rs5194	23	115304830	*AGTR2*	A/G	0.38	-0.67	0.17	7.55E-05	1.92E-04
rs11091046	23	115305126	*AGTR2*	A/C	0.38	-0.68	0.17	5.39E-05	1.32E-04

CA=Coded allele; CAF= Coded allele frequency; Chr=chromosome; OA=Other allele

Table S12. Suggestive loci identified in the discovery stage 1 df interaction and 2df joint tests with obesity (P <1×10^{-4})

Variant	Chr	Position	Gene	CA/OA	CAF	1 df interaction test			***2 df joint test P-value***
						**Beta**	**SE**	***P-value***	
rs1801133	1	11856378	*MTHFR*	A/G	0.55	-0.06	0.21	7.81E-01	5.66E-07
chr1_156647165	1	156647165	*NES*	CG/C	0.06	0.02	0.45	9.68E-01	4.30E-05
chr2_102508323	2	102508323	*MAP4K4*	TG/T	0.08	-0.49	0.41	2.34E-01	6.00E-05
rs4512220	6	44923138	*SUPT3H*	C/G	0.28	-0.30	0.22	1.76E-01	1.31E-05
chr8_1818398	8	1818398	*ARHGEF10*	G/GA	0.06	0.75	0.60	2.08E-01	1.84E-05
chr8_27403032	8	27403032	*EPHX2*	GC/G	0.04	0.19	0.63	7.63E-01	2.12E-06
chr11_61537791	11	61537791	*MYRF*	GC/G	0.03	-0.76	0.73	3.01E-01	3.47E-05
chr14_90864456	14	90864456	*CALM1*	C/CG	0.38	-0.61	0.23	8.03E-03	2.29E-05
chr16_73095268	16	73095268	*ZFHX3; HCCAT5*	G/GC	0.03	-1.29	0.65	4.60E-02	2.45E-05
chr19_51323750	19	51323750	*KLK1*	G/GAT	0.06	-0.45	0.44	3.01E-01	6.86E-05

CA=Coded allele; CAF= Coded allele frequency; Chr=chromosome; OA=Other allele

Table S13. Suggestive loci identified in the discovery stage 1 df interaction and 2df joint tests with history of hypertension (P <1×10^{-4})

Variant	Chr	Position	Gene	CA/OA	CAF	1 df interaction test			***2 df joint test P-value***
						**Beta**	**SE**	***P-value***	
rs225125	1	8087006	*ERRFI1*	G/A	0.02	-0.90	0.72	2.11E-01	2.05E-06
chr2_102508323	2	102508323	*MAP4K4*	TG/T	0.08	0.69	0.48	1.46E-01	1.06E-06
chr5_131718063	5	131718063	*SLC22A5*	CA/C	0.04	-0.90	0.45	4.65E-02	1.53E-05
rs72456916	6	44795920	*SUPT3H*	A/AAAGTT	0.37	-0.74	0.18	6.15E-05	6.89E-05
rs9369514	6	44797271	*SUPT3H*	C/A	0.37	-0.72	0.18	8.02E-05	9.42E-05
rs4512220	6	44923138	*SUPT3H*	C/G	0.28	-0.59	0.19	2.22E-03	2.29E-05
chr8_27403032	8	27403032	*EPHX2*	GC/G	0.04	0.49	0.66	4.58E-01	1.61E-05
chr16_73095268	16	73095268	*ZFHX3; HCCAT5*	G/GC	0.03	1.03	1.06	3.33E-01	6.78E-05
chr19_51323750	19	51323750	*KLK1*	G/GAT	0.06	0.43	0.49	3.79E-01	3.32E-05

CA=Coded allele; CAF= Coded allele frequency; Chr=chromosome; OA=Other allele

Table S14. Suggestive loci identified in the discovery stage 1 df interaction and 2df joint tests with history of hyperlipidemia ($P < 1\times10^{-4}$)

Variant	Chr	Position	Gene	CA/OA	CAF	1 df interaction test			*2 df joint test P-value*
						Beta	SE	*P-value*	
rs1801133	1	11856378	*MTHFR*	A/G	0.55	-0.40	0.35	2.64E-01	5.75E-08
chr1_156647165	1	156647165	*NES*	CG/C	0.06	1.28	1.09	2.41E-01	4.90E-05
chr10_104718215	10	104718215	*CNNM2*	T/TG	0.04	0.49	1.11	6.61E-01	7.50E-05
chr16_73095267	16	73095267	*ZFHX3; HCCAT5*	T/TGCC	0.06	0.37	0.84	6.63E-01	2.82E-05
chr19_51323750	19	51323750	*KLK1*	G/GAT	0.06	-0.92	0.80	2.49E-01	3.21E-06

CA=Coded allele; CAF= Coded allele frequency; Chr=chromosome; OA=Other allele

Table S15. Loci achieving P<1×10^{-4} in the discovery stage main effects analyses and selected for replication study

Variant	Chr	Position	Gene	CA/OA	CAF	Stage	Beta	SE	*P* value
rs1801133	1	11856378	*MTHFR*	A/G	0.55	Discovery	0.37	0.07	1.54E-08
					0.48	Replication	0.15	0.03	6.56E-07
						Meta	0.18	0.03	7.54E-12
rs174535	11	61551356	*MYRF*	C/T	0.34	Discovery	-0.27	0.07	1.35E-04
					0.37	Replication	-0.17	0.03	3.61E-08
						Meta	-0.18	0.03	4.81E-11
rs174545	11	61569306	*FADS1*	G/C	0.33	Discovery	-0.27	0.07	1.48E-04
					0.37	Replication	-0.17	0.03	1.98E-08
						Meta	-0.19	0.03	2.78E-11
rs3834458	11	61594920	*FADS2*	C/CT	0.33	Discovery	-0.27	0.07	1.85E-04
					0.36	Replication	-0.17	0.03	1.45E-08
						Meta	-0.19	0.03	2.30E-11
rs3782886	12	112110489	*BRAP*	C/T	0.16	Discovery	-0.09	0.09	2.88E-01
					0.20	Replication	-0.20	0.04	1.15E-07
						Meta	-0.18	0.03	1.22E-07
rs671	12	112241766	*ALDH2*	A/G	0.16	Discovery	-0.10	0.09	2.48E-01
					0.19	Replication	-0.21	0.04	2.44E-08
						Meta	-0.19	0.03	2.36E-08
rs78069066	12	112337924	*ADAM1A*	A/G	0.16	Discovery	-0.10	0.09	2.38E-01
					0.20	Replication	-0.19	0.04	1.60E-07
						Meta	-0.18	0.03	1.28E-07

CA=Coded allele; CAF= Coded allele frequency; Chr=chromosome; OA=Other allele

Table S16. Loci achieving P<1×10^{-4} in the discovery stage 1 df interaction or 2 df joint tests and selected for replication study

Environmental Variable	Variant	Chr	Position	Gene	CA/OA	CAF	Stage	Variant-environment interaction			2df joint test *P*-value
								Beta	SE	*P*-value	
Sex	rs1801133	1	11856378	*MTHFR*	A/G	0.55	Discovery	0.12	0.13	3.69E-01	7.91E-08
						0.48	Replication	-0.11	0.06	6.36E-02	7.84E-07
							Meta-analysis	-0.07	0.05	1.81E-01	2.72E-11
Body-mass index	rs1801133	1	11856378	*MTHFR*	A/G	0.55	Discovery	-0.004	0.02	8.43E-01	3.51E-07
						0.48	Replication	-0.01	0.01	3.79E-01	4.12E-11
							Meta-analysis	-0.009	0.01	3.83E-01	3.92E-17
Fasting plasma glucose	rs1801133	1	11856378	*MTHFR*	A/G	0.55	Discovery	-0.05	0.05	3.23E-01	2.79E-07
						0.48	Replication	0.002	0.018	9.05E-01	8.55E-10
							Meta-analysis	-0.004	0.017	8.37E-01	1.08E-15
	rs146404695	5	58571684	*PDE4D*	C/T	0.02	Discovery	-0.37	0.09	1.61E-05	1.89E-05
						0.03	Replication	-0.05	0.05	2.53E-01	7.08E-02
							Meta-analysis	-0.13	0.04	2.05E-03	5.98E-03
	rs144146728	6	49403301	*MUT*	T/C	0.03	Discovery	-0.37	0.09	5.00E-05	1.99E-04
						0.02	Replication	-0.02	0.07	7.76E-01	9.01E-01
							Meta-analysis	-0.14	0.05	9.34E-03	2.56E-02
	rs7075480	10	100176627	*HPS1*	A/G	0.06	Discovery	-0.31	0.08	6.89E-05	3.40E-04
						0.07	Replication	0.03	0.04	4.36E-01	6.82E-01
							Meta-analysis	-0.04	0.03	2.93E-01	5.66E-01
	rs2250526	17	57951973	*TUBD1*	A/G	0.42	Discovery	-0.20	0.05	4.29E-05	4.83E-05
						0.41	Replication	0.03	0.02	1.52E-01	2.39E-01

							Meta-analysis	-0.002	0.02	9.01E-01	9.77E-01
	rs41345851	20	23063779	*CD93*	T/C	0.15	Discovery	-0.23	0.05	1.38E-05	7.82E-05
						0.15	Replication	0.01	0.03	7.49E-01	9.50E-01
							Meta-analysis	-0.04	0.02	1.13E-01	2.84E-01
	rs41418351	20	23064912	*CD93*	C/T	0.15	Discovery	-0.21	0.05	8.44E-05	4.39E-04
						0.15	Replication	0.004	0.025	8.74E-01	9.26E-01
							Meta-analysis	-0.03	0.02	1.27E-01	2.93E-01
HDL-cholesterol	rs1801133	1	11856378	*MTHFR*	A/G	0.55	Discovery	-0.24	0.19	1.90E-01	1.64E-07
						0.48	Replication	0.10	0.09	2.20E-01	4.68E-07
							Meta-analysis	0.04	0.08	5.69E-01	1.24E-11
	rs2287339	2	53992593	*ASB3; GPR75-ASB3*	T/A	0.25	Discovery	0.71	0.18	5.19E-05	7.50E-05
						0.26	Replication	0.12	0.10	2.27E-01	1.44E-01
							Meta-analysis	0.26	0.09	2.38E-03	1.50E-02
	rs76328173	2	54087470	*GPR75; GPR75-ASB3*	A/G	0.25	Discovery	0.73	0.18	4.40E-05	3.86E-05
						0.27	Replication	0.16	0.10	1.10E-01	6.88E-02
							Meta-analysis	0.29	0.09	7.89E-04	5.94E-03
	rs1194182	7	80231504	*CD36*	G/C	0.37	Discovery	-0.86	0.19	4.66E-06	2.70E-05
						0.36	Replication	-0.09	0.09	3.15E-01	4.81E-01
							Meta-analysis	-0.23	0.08	4.00E-03	1.25E-02
	rs73167652	7	150700637	*NOS3*	G/A	0.08	Discovery	1.24	0.27	3.45E-06	9.39E-06
						0.08	Replication	0.26	0.16	9.76E-02	2.54E-01
							Meta-analysis	0.52	0.14	1.41E-04	4.34E-04
	rs2916715	8	6357307	*ANGPT2*	C/T	0.10	Discovery	0.99	0.25	7.67E-05	1.26E-05
						0.09	Replication	-0.29	0.16	7.15E-02	1.96E-01

							Meta-analysis	0.09	0.14	5.30E-01	3.39E-01
	rs1961222	8	6377433	*ANGPT2*	T/C	0.08	Discovery	1.00	0.26	1.01E-04	3.76E-05
						0.07	Replication	-0.25	0.18	1.68E-01	3.55E-01
							Meta-analysis	0.16	0.15	2.68E-01	1.46E-01
	rs328	8	19819724	*LPL*	G/C	0.08	Discovery	1.12	0.28	5.98E-05	4.68E-05
						0.08	Replication	-0.02	0.15	8.73E-01	9.79E-01
							Meta-analysis	0.24	0.13	7.28E-02	5.56E-02
	rs1803924	8	19823674	*LPL*	T/C	0.08	Discovery	1.09	0.28	8.30E-05	6.88E-05
						0.08	Replication	-0.04	0.15	7.85E-01	9.63E-01
							Meta-analysis	0.22	0.13	9.42E-02	7.81E-02
	rs3735964	8	19824045	*LPL*	A/C	0.08	Discovery	1.09	0.28	8.06E-05	6.52E-05
						0.08	Replication	-0.04	0.15	7.77E-01	9.60E-01
							Meta-analysis	0.22	0.13	9.54E-02	7.78E-02
	rs1059611	8	19824563	*LPL*	C/T	0.08	Discovery	1.10	0.28	7.13E-05	6.11E-05
						0.08	Replication	-0.03	0.15	8.38E-01	9.20E-01
							Meta-analysis	0.23	0.13	8.15E-02	5.16E-02
	rs10645926	8	19824626	*LPL*	CTT/C	0.08	Discovery	1.10	0.28	7.05E-05	5.59E-05
						0.08	Replication	-0.07	0.16	6.36E-01	6.59E-01
							Meta-analysis	0.21	0.14	1.26E-01	1.88E-01
	rs15285	8	19824667	*LPL*	T/C	0.08	Discovery	1.07	0.28	1.50E-04	5.36E-05
						0.19	Replication	0.11	0.11	3.09E-01	5.72E-01
							Meta-analysis	0.24	0.10	1.96E-02	2.25E-02
	rs12573199	10	104848844	*NT5C2*	T/A	0.08	Discovery	1.04	0.27	9.53E-05	4.93E-04
						0.07	Replication	-0.19	0.17	2.73E-01	5.25E-01

							Meta-analysis	0.17	0.14	2.33E-01	4.79E-01
	rs12573200	10	104848855	*NT5C2*	T/A	0.08	Discovery	1.04	0.27	9.54E-05	4.89E-04
						0.07	Replication	-0.15	0.17	3.65E-01	6.32E-01
							Meta-analysis	0.19	0.14	1.79E-01	3.87E-01
	rs12573221	10	104849144	*NT5C2*	C/A	0.08	Discovery	1.07	0.27	6.41E-05	3.39E-04
						0.07	Replication	-0.19	0.17	2.70E-01	5.28E-01
							Meta-analysis	0.18	0.14	2.18E-01	4.62E-01
	rs174535	11	61551356	*MYRF*	C/T	0.34	Discovery	-0.53	0.20	1.00E-02	4.37E-06
						0.37	Replication	-0.02	0.09	7.97E-01	9.18E-09
							Meta-analysis	-0.11	0.08	1.97E-01	1.24E-12
	rs174545	11	61569306	*FADS1*	G/C	0.33	Discovery	-0.51	0.21	1.44E-02	9.95E-06
						0.37	Replication	-0.11	0.09	2.17E-01	2.07E-09
							Meta-analysis	-0.18	0.08	3.34E-02	1.67E-13
	rs3834458	11	61594920	*FADS2*	C/CT	0.33	Discovery	-0.51	0.21	1.38E-02	1.25E-05
						0.36	Replication	-0.13	0.09	1.68E-01	1.13E-09
							Meta-analysis	-0.19	0.08	2.36E-02	1.70E-12
	rs1061228	16	88782079	*PIEZO1*	A/G	0.15	Discovery	0.76	0.20	9.51E-05	2.02E-04
						0.14	Replication	0.17	0.12	1.61E-01	8.82E-02
							Meta-analysis	0.34	0.10	1.10E-03	4.83E-04
	rs6105	18	61565062	*SERPINB2*	G/C	0.07	Discovery	1.11	0.27	2.77E-05	6.94E-05
						0.06	Replication	-0.02	0.18	9.31E-01	4.58E-01
							Meta-analysis	0.33	0.15	2.51E-02	1.77E-02
LDL-cholesterol	rs1801133	1	11856378	*MTHFR*	A/G	0.55	Discovery	0.01	0.07	8.85E-01	5.17E-07
						0.48	Replication	-0.04	0.03	9.47E-02	2.24E-07

							Meta-analysis	-0.04	0.03	1.32E-01	7.50E-12
Triglycerides	rs1801133	1	11856378	*MTHFR*	A/G	0.55	Discovery	0.11	0.13	4.28E-01	1.98E-07
						0.48	Replication	-0.09	0.05	5.62E-02	9.04E-08
							Meta-analysis	-0.07	0.05	1.30E-01	2.31E-12
	rs174535	11	61551356	*MYRF*	C/T	0.34	Discovery	-0.05	0.14	7.25E-01	8.15E-05
						0.37	Replication	-0.04	0.05	4.78E-01	8.87E-08
							Meta-analysis	-0.04	0.05	4.30E-01	3.07E-11
Alcohol drinking	rs1801133	1	11856378	*MTHFR*	A/G	0.55	Discovery	-0.33	0.14	1.69E-02	6.22E-09
						0.48	Replication	0.24	0.07	2.01E-04	4.46E-09
							Meta-analysis	0.14	0.06	1.87E-02	2.50E-12
	rs4148217	2	44099433	*ABCG8*	A/C	0.12	Discovery	0.85	0.21	6.67E-05	1.61E-04
						0.11	Replication	0.08	0.10	4.40E-01	4.15E-01
							Meta-analysis	0.23	0.09	1.50E-02	6.54E-02
	rs3782886	12	112110489	*BRAP*	C/T	0.16	Discovery	-0.88	0.23	1.19E-04	6.66E-05
						0.20	Replication	-0.68	0.10	1.72E-12	2.68E-19
							Meta-analysis	-0.71	0.09	1.33E-15	2.51E-23
	rs671	12	112241766	*ALDH2*	A/G	0.16	Discovery	-0.95	0.23	5.47E-05	3.06E-05
						0.19	Replication	-0.73	0.10	9.20E-14	4.23E-21
							Meta-analysis	-0.76	0.09	3.39E-17	2.63E-25
	rs78069066	12	112337924	*ADAM1A*	A/G	0.16	Discovery	-0.83	0.23	2.45E-04	9.97E-05
						0.20	Replication	-0.68	0.10	1.20E-12	2.40E-19
							Meta-analysis	-0.71	0.09	1.55E-15	2.66E-23
	rs2664593	17	4545132	*ALOX15*	G/C	0.19	Discovery	0.69	0.18	9.30E-05	2.86E-04
						0.19	Replication	0.07	0.08	3.92E-01	4.62E-01

							Meta-analysis	0.19	0.08	1.39E-02	1.99E-02
Cigarette smoking	rs1801133	1	11856378	*MTHFR*	A/G	0.55	Discovery	-0.22	0.14	1.12E-01	3.18E-08
						0.48	Replication	0.20	0.06	9.81E-04	2.62E-08
							Meta-analysis	0.13	0.06	1.80E-02	7.04E-12
Obesity	rs1801133	1	11856378	*MTHFR*	A/G	0.55	Discovery	-0.06	0.21	7.81E-01	5.66E-07
						0.48	Replication	-0.10	0.11	3.91E-01	4.59E-11
							Meta-analysis	-0.09	0.10	3.76E-01	8.39E-17
History of hypertension	rs72456916	6	44795920	*SUPT3H*	A/AAAGTT	0.37	Discovery	-0.74	0.18	6.15E-05	6.89E-05
						0.37	Replication	0.06	0.07	4.08E-01	4.28E-01
							Meta-analysis	-0.04	0.07	5.14E-01	2.24E-01
	rs9369514	6	44797271	*SUPT3H*	C/A	0.37	Discovery	-0.72	0.18	8.02E-05	9.42E-05
						0.35	Replication	0.06	0.07	4.07E-01	4.04E-01
							Meta-analysis	-0.04	0.07	5.28E-01	2.07E-01
History of hyperlipidemia	rs1801133	1	11856378	*MTHFR*	A/G	0.55	Discovery	-0.40	0.35	2.64E-01	5.75E-08
						0.48	Replication	-0.34	0.16	3.66E-02	6.13E-04
							Meta-analysis	-0.35	0.15	1.80E-02	7.17E-09

CA=Coded allele; CAF= Coded allele frequency; Chr=chromosome; OA=Other allele

Table S17. Association between identified variants and stroke according to drinking status and sex.

Variant	Chr	Position (Build 37)	Gene	CA/OA	CAF	Stage	Drinkers					Non-drinkers			
							Male (n=2784)		Female (n=727)			Male (n=3719)		Female (n=4379)	
							OR (95% CI)	*P* value	OR (95% CI)	*P* value		OR (95% CI)	*P* value	OR (95% CI)	*P* value
rs3782886*	12	112110489	*BRAP*	C/T	0.16	Discovery	0.34 (0.21, 0.55)	1.08E-05	0.70 (0.19, 2.60)	5.93E-01		0.84 (0.64, 1.11)	2.15E-01	1.26 (0.94, 1.71)	1.27E-01
					0.20	Replication	0.55 (0.45, 0.67)	4.76E-09	1.39 (0.85, 2.29)	1.91E-01		0.81 (0.72, 0.92)	8.39E-04	0.93 (0.82, 1.04)	1.92E-01
						Meta	0.51 (0.42, 0.61)	1.20E-12	1.28 (0.80, 2.03)	3.01E-01		0.82 (0.73, 0.91)	3.78E-04	0.96 (0.86, 1.07)	5.03E-01
rs671*	12	112241766	*ALDH2*	A/G	0.16	Discovery	0.29 (0.17, 0.47)	1.17E-06	0.97 (0.31, 3.01)	9.59E-01		0.86 (0.65, 1.13)	2.78E-01	1.26 (0.93, 1.72)	1.39E-01
					0.19	Replication	0.52 (0.42, 0.63)	2.77E-10	1.38 (0.84, 2.26)	2.09E-01		0.81 (0.72, 0.92)	9.04E-04	0.92 (0.82, 1.03)	1.61E-01
						Meta	0.47 (0.39, 0.57)	1.58E-14	1.30 (0.82, 2.05)	2.59E-01		0.82 (0.73, 0.92)	5.16E-04	0.96 (0.86, 1.07)	4.32E-01
rs78069066*	12	112337924	*ADAM1A*	A/G	0.16	Discovery	0.34 (0.21, 0.55)	1.08E-05	0.95 (0.31, 2.96)	9.32E-01		0.83 (0.63, 1.09)	1.78E-01	1.26 (0.93, 1.71)	1.37E-01
					0.20	Replication	0.54 (0.44, 0.66)	2.31E-09	1.40 (0.85, 2.30)	1.85E-01		0.82 (0.72, 0.93)	1.31E-03	0.92 (0.82, 1.04)	1.81E-01
						Meta	0.50 (0.42, 0.61)	5.35E-13	1.31 (0.83, 2.07)	2.38E-01		0.82 (0.73, 0.92)	4.95E-04	0.96 (0.86, 1.07)	4.72E-01

CA=Coded allele; CAF= Coded allele frequency; Chr=chromosome; OA=Other allele

Table S18. Comparison of variants main effects under additive, dominant and recessive models.

Variant	Chr	Position (Build 37)	Gene	CA/OA	CAF	Stage	Additive model		Dominant model		Recessive model	
							OR (95% CI)	*P* value	OR (95% CI)	*P* value	OR (95% CI)	*P* value
rs1801133	1	11856378	*MTHFR*	A/G	0.55	Discovery	1.45 (1.26, 1.66)	1.54E-08	1.57 (1.29, 1.91)	6.46E-06	1.75 (1.39, 2.20)	1.63E-06
					0.48	Replication	1.16 (1.10, 1.23)	6.56E-07	1.19 (1.08, 1.30)	2.26E-04	1.25 (1.13, 1.37)	8.47E-06
						Meta-analysis	1.20 (1.13, 1.27)	7.54E-12	1.25 (1.15, 1.36)	1.62E-07	1.31 (1.20, 1.43)	2.38E-09
rs174535[*]	11	61551356	*MYRF*	C/T	0.34	Discovery	0.76 (0.67, 0.88)	1.35E-04	0.76 (0.63, 0.92)	3.71E-03	0.59 (0.44, 0.80)	5.09E-04
					0.37	Replication	0.84 (0.80, 0.89)	3.61E-08	0.80 (0.74, 0.87)	3.35E-07	0.80 (0.71, 0.90)	2.24E-04
						Meta-analysis	0.84 (0.79, 0.89)	4.81E-11	0.80 (0.74, 0.86)	5.00E-09	0.77 (0.69, 0.86)	2.31E-06
rs174545[*]	11	61569306	*FADS1*	G/C	0.33	Discovery	0.76 (0.67, 0.88)	1.48E-04	0.77 (0.64, 0.92)	4.74E-03	0.57 (0.42, 0.78)	3.70E-04
					0.37	Replication	0.84 (0.80, 0.89)	1.98E-08	0.80 (0.73, 0.87)	1.14E-07	0.80 (0.71, 0.90)	3.11E-04
						Meta-analysis	0.83 (0.78, 0.88)	2.78E-11	0.79 (0.74, 0.86)	1.07E-08	0.77 (0.69, 0.86)	3.14E-06
rs3834458[*]	11	61594920	*FADS2*	C/CT	0.33	Discovery	0.76 (0.67, 0.88)	1.85E-04	0.77 (0.65, 0.94)	8.00E-03	0.56 (0.41, 0.76)	2.05E-04
					0.36	Replication	0.84 (0.80, 0.89)	1.45E-08	0.80 (0.73, 0.87)	9.95E-08	0.80 (0.71, 0.90)	2.29E-04
						Meta-analysis	0.83 (0.78, 0.88)	2.30E-11	0.79 (0.74, 0.86)	2.69E-09	0.76 (0.68, 0.85)	1.70E-06
rs3782886[†]	12	112110489	*BRAP*	C/T	0.16	Discovery	0.91 (0.77, 1.09)	2.88E-01	0.90 (0.74, 1.10)	3.10E-01	0.86 (0.51, 1.43)	5.59E-01
					0.20	Replication	0.82 (0.76, 0.89)	1.15E-07	0.81 (0.74, 0.88)	1.40E-06	0.69 (0.56, 0.85)	4.79E-04

						Meta-analysis	0.84 (0.79, 0.89)	1.22E-07		0.82 (0.76, 0.89)	1.36E-06		0.71 (0.58, 0.86)	5.56E-04
rs671[†]	12	112241766	*ALDH2*	A/G	0.16	Discovery	0.90 (0.76, 1.08)	2.48E-01		0.89 (0.73, 1.09)	2.66E-01		0.85 (0.50, 1.43)	5.35E-01
					0.19	Replication	0.81 (0.75, 0.88)	2.44E-08		0.80 (0.73, 0.87)	2.40E-07		0.68 (0.55, 0.85)	5.48E-04
						Meta-analysis	0.83 (0.78, 0.88)	2.36E-08		0.81 (0.75, 0.88)	2.21E-07		0.71 (0.58, 0.86)	5.94E-04
rs78069066[†]	12	112337924	*ADAM1A*	A/G	0.16	Discovery	0.90 (0.76, 1.08)	2.38E-01		0.88 (0.72, 1.08)	2.21E-01		0.90 (0.57, 1.50)	6.89E-01
					0.20	Replication	0.83 (0.76, 0.89)	1.60E-07		0.81 (0.75, 0.89)	2.33E-06		0.68 (0.55, 0.84)	3.63E-04
						Meta-analysis	0.84 (0.79, 0.89)	1.28E-07		0.82 (0.76, 0.89)	1.44E-06		0.71 (0.59, 0.86)	5.55E-04

CA=Coded allele; CAF= Coded allele frequency; Chr=chromosome; OA=Other allele; OR=odds ratio

[*] rs174535, rs174545, and rs3834458 were highly correlated, smallest R^2=0.99

[†] rs3782886, rs671, and rs78069066 were highly correlated, smallest R^2=0.98

Table S19. Comparison of variants interaction effects under additive, dominant and recessive models.

Variant	Chr	Position (Build 37)	Gene	CA/OA	CAF	Stage	Drinkers			Non-drinkers			1df Interaction *P* value
							OR	95% CI	*P* value	OR	95% CI	*P* value	
Additive model													
rs3782886*	12	112110489	*BRAP*	C/T	0.16	Discovery	0.37	(0.24, 0.59)	2.06E-05	1.01	(0.82, 1.23)	9.60E-01	1.19E-04
					0.20	Replication	0.61	(0.51, 0.74)	1.93E-07	0.88	(0.81, 0.96)	2.68E-03	1.72E-12
						Meta	0.57	(0.48, 0.68)	1.25E-10	0.90	(0.83, 0.97)	5.96E-03	1.33E-15
rs671*	12	112241766	*ALDH2*	A/G	0.16	Discovery	0.34	(0.21, 0.54)	6.47E-06	1.01	(0.83, 1.24)	9.02E-01	5.47E-05
					0.19	Replication	0.58	(0.48, 0.70)	1.39E-08	0.88	(0.80, 0.95)	2.27E-03	9.20E-14
						Meta	0.54	(0.45, 0.64)	3.55E-12	0.89	(0.83, 0.97)	5.60E-03	3.39E-17
rs78069066*	12	112337924	*ADAM1A*	A/G	0.16	Discovery	0.39	(0.25, 0.62)	4.36E-05	0.99	(0.81, 1.22)	9.42E-01	2.45E-04
					0.20	Replication	0.61	(0.50, 0.73)	1.14E-07	0.88	(0.81, 0.96)	3.19E-03	1.20E-12
						Meta	0.57	(0.48, 0.67)	1.01E-10	0.90	(0.83, 0.97)	5.99E-03	1.55E-15
Dominant model													
rs3782886*	12	112110489	*BRAP*	C/T	0.16	Discovery	0.37	(0.23, 0.60)	4.26E-05	1.02	(0.80, 1.30)	8.81E-01	2.27E-04
					0.20	Replication	0.58	(0.47, 0.71)	2.93E-07	0.88	(0.79, 0.97)	1.15E-02	6.42E-12
						Meta	0.54	(0.45, 0.65)	2.21E-10	0.90	(0.82, 0.99)	2.28E-02	7.55E-15
rs671*	12	112241766	*ALDH2*	A/G	0.16	Discovery	0.32	(0.19, 0.52)	4.47E-06	1.03	(0.81, 1.31)	8.30E-01	4.80E-05
					0.19	Replication	0.55	(0.44, 0.67)	1.85E-08	0.87	(0.78, 0.96)	7.64E-03	3.51E-13
						Meta	0.50	(0.41, 0.61)	2.96E-12	0.89	(0.81, 0.98)	1.72E-02	1.04E-16
rs78069066*	12	112337924	*ADAM1A*	A/G	0.16	Discovery	0.37	(0.23, 0.60)	4.1E-05	0.99	(0.78, 1.26)	9.50E-01	3.25E-04
					0.20	Replication	0.57	(0.47, 0.71)	1.60E-07	0.88	(0.80, 0.98)	1.56E-02	3.60E-12
						Meta	0.53	(0.44, 0.65)	1.11E-10	0.90	(0.82, 0.99)	2.39E-02	5.55E-15
Recessive model													
rs3782886*	12	112110489	*BRAP*	C/T	0.16	Discovery[a]	NA	NA	NA	0.94	(0.54, 1.65)	8.35E-01	NA
					0.20	Replication	0.46	(0.24, 0.85)	1.41E-02	0.75	(0.59, 0.95)	1.60E-02	3.57E-04

						Meta	NA	NA	NA	0.77	(0.62, 0.96)	2.14E-02	NA
rs671*	12	112241766	*ALDH2*	A/G	0.16	Discovery	0.22	(0.03, 1.85)	1.62E-01	0.95	(0.53, 1.71)	8.69E-01	1.80E-01
					0.19	Replication	0.42	(0.22, 0.81)	9.64E-03	0.76	(0.60, 0.97)	2.44E-02	2.35E-04
						Meta	0.40	(0.21, 0.75)	3.94E-03	0.78	(0.63, 0.98)	3.21E-02	9.12E-05
rs78069066*	12	112337924	*ADAM1A*	A/G	0.16	Discovery	0.22	(0.03, 1.85)	1.63E-01	0.98	(0.56, 1.74)	9.56E-01	1.61E-01
					0.20	Replication	0.45	(0.24, 0.85)	1.38E-02	0.74	(0.59, 0.94)	1.19E-02	4.14E-04
						Meta	0.43	(0.23, 0.78)	5.85E-03	0.77	(0.62, 0.96)	1.91E-02	1.52E-04

CA=Coded allele; CAF= Coded allele frequency; Chr=chromosome; OA=Other allele; OR=odds ratio

[a]: For rs3782886, when using recessive model, there were no stroke cases with two mutations.

* rs3782886, rs671, and rs78069066 were highly correlated, smallest R^2=0.98

Table S20. Comparison of identified variants 1 df interaction effects using standard error and robust standard error.

Variant	Chr	Position (Build 37)	Gene	CA/OA	CAF	Stage	1df Interaction *P* value using standard error	1df Interaction *P* value using robust standard error
rs3782886	12	112110489	*BRAP*	C/T	0.16	Discovery	1.19E-04	3.83E-05
					0.20	Replication	1.72E-12	3.53E-13
						Meta	1.33E-15	8.91E-17
rs671	12	112241766	*ALDH2*	A/G	0.16	Discovery	5.47E-05	1.55E-05
					0.19	Replication	9.20E-14	1.44E-14
						Meta	3.39E-17	1.54E-18
rs78069066	12	112337924	*ADAM1A*	A/G	0.16	Discovery	2.45E-04	1.04E-04
					0.20	Replication	1.20E-12	2.31E-13
						Meta	1.55E-15	1.30E-16

CA=Coded allele; CAF= Coded allele frequency; Chr=chromosome; OA=Other allele

Table S21. Comparison of identified variants 2 df joint effects using standard error and robust standard error.

**Environmental Variable**	Variant	Chr	Position (Build 37)	Gene	CA/OA	CAF	Stage	2df Joint *P* value using standard error	2df Joint *P* value using robust standard error
Body-mass index	rs1801133	1	11856378	*MTHFR*	A/G	0.55	Discovery	3.51E-07	2.17E-07
						0.48	Replication	4.12E-11	6.78E-11
							Meta-analysis	3.92E-17	4.90E-17
Fasting plasma glucose	rs1801133	1	11856378	*MTHFR*	A/G	0.55	Discovery	2.79E-07	2.68E-07
						0.48	Replication	8.55E-10	1.26E-09
							Meta-analysis	1.08E-15	2.94E-15
HDL-cholesterol	rs1801133	1	11856378	*MTHFR*	A/G	0.55	Discovery	1.64E-07	1.10E-07
						0.48	Replication	4.68E-07	8.64E-07
							Meta-analysis	1.24E-11	1.49E-11
	rs174535	11	61551356	*MYRF*	C/T	0.34	Discovery	4.37E-06	1.65E-05
						0.37	Replication	9.18E-09	6.68E-09
							Meta-analysis	1.24E-12	1.08E-12
	rs174545	11	61569306	*FADS1*	G/C	0.33	Discovery	9.95E-06	3.36E-05
						0.37	Replication	2.07E-09	2.59E-09
							Meta-analysis	1.67E-13	3.81E-13
	rs3834458	11	61594920	*FADS2*	C/CT	0.33	Discovery	1.25E-05	4.58E-05
						0.36	Replication	1.13E-09	1.60E-09
							Meta-analysis	1.70E-12	2.88E-13
LDL-cholesterol	rs1801133	1	11856378	*MTHFR*	A/G	0.55	Discovery	5.17E-07	3.83E-07
						0.48	Replication	2.24E-07	2.19E-07
							Meta-analysis	7.50E-12	4.78E-12
Triglycerides	rs1801133	1	11856378	*MTHFR*	A/G	0.55	Discovery	1.98E-07	2.04E-07
						0.48	Replication	9.04E-08	1.31E-08

							Meta-analysis	2.31E-12	4.27E-13
	rs174535	11	61551356	*MYRF*	C/T	0.34	Discovery	8.15E-05	6.54E-05
						0.37	Replication	8.87E-08	1.13E-07
							Meta-analysis	3.07E-11	5.93E-11
Alcohol drinking	rs1801133	1	11856378	*MTHFR*	A/G	0.55	Discovery	6.22E-09	1.33E-08
						0.48	Replication	4.46E-09	1.34E-09
							Meta-analysis	2.50E-12	2.06E-11
	rs3782886	12	112110489	*BRAP*	C/T	0.16	Discovery	6.66E-05	1.04E-06
						0.20	Replication	2.68E-19	5.91E-23
							Meta	2.51E-23	6.83E-26
	rs671	12	112241766	*ALDH2*	A/G	0.16	Discovery	3.06E-05	2.82E-07
						0.19	Replication	4.23E-21	1.89E-25
							Meta	2.63E-25	7.69E-32
	rs78069066	12	112337924	*ADAM1A*	A/G	0.16	Discovery	9.97E-05	2.40E-06
						0.20	Replication	2.40E-19	4.81E-23
							Meta	2.67E-23	1.43E-28
Cigarette smoking	rs1801133	1	11856378	*MTHFR*	A/G	0.55	Discovery	3.18E-08	3.98E-08
						0.48	Replication	2.62E-08	1.01E-08
							Meta-analysis	7.04E-12	1.98E-12
Obesity	rs1801133	1	11856378	*MTHFR*	A/G	0.55	Discovery	5.66E-07	4.08E-07
						0.48	Replication	4.59E-11	9.72E-11
							Meta-analysis	8.39E-17	1.12E-16
History of hyperlipidemia	rs1801133	1	11856378	*MTHFR*	A/G	0.55	Discovery	5.75E-08	3.99E-08
						0.48	Replication	6.13E-04	6.47E-04
							Meta-analysis	7.17E-09	8.17E-09

CA=Coded allele; CAF= Coded allele frequency; Chr=chromosome; OA=Other allele

Figure S1. Circular Manhattan plots displaying variants achieving suggestive significance in the 1df interaction (inner circle) and 2df joint tests (outer circle). Red dashed lines indicate suggestive significance ($P<1.0\times10^{-4}$) in the discovery stage

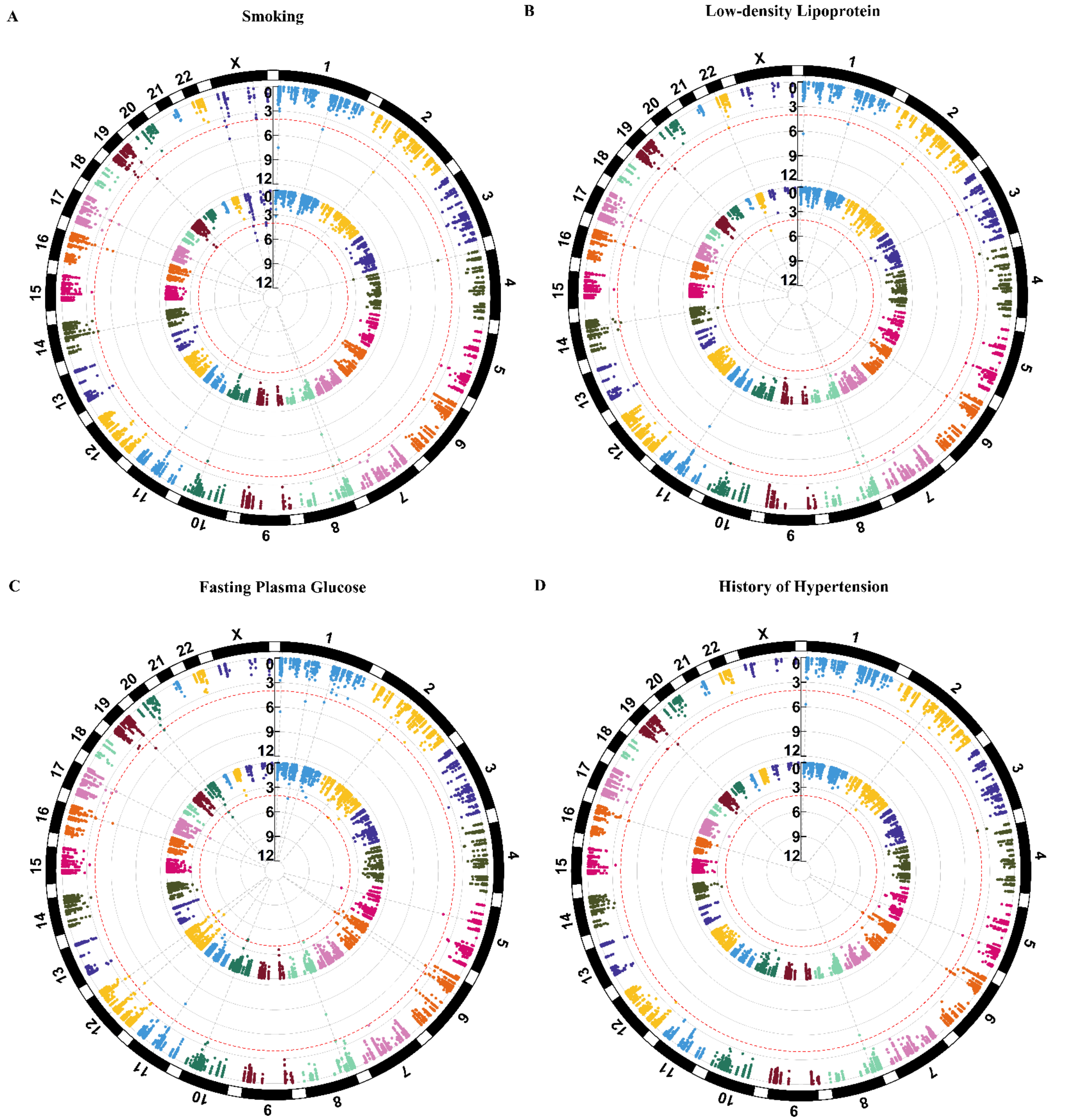

Figure S2. Circular Manhattan plots displaying variants achieving suggestive significance in only the discovery stage 2df joint test. Inner circle displays 1 df interaction tests and outer circle displays 2 df joint tests. Red dashed lines indicate suggestive significance ($P<1.0\times10^{-4}$) in the discovery stage.

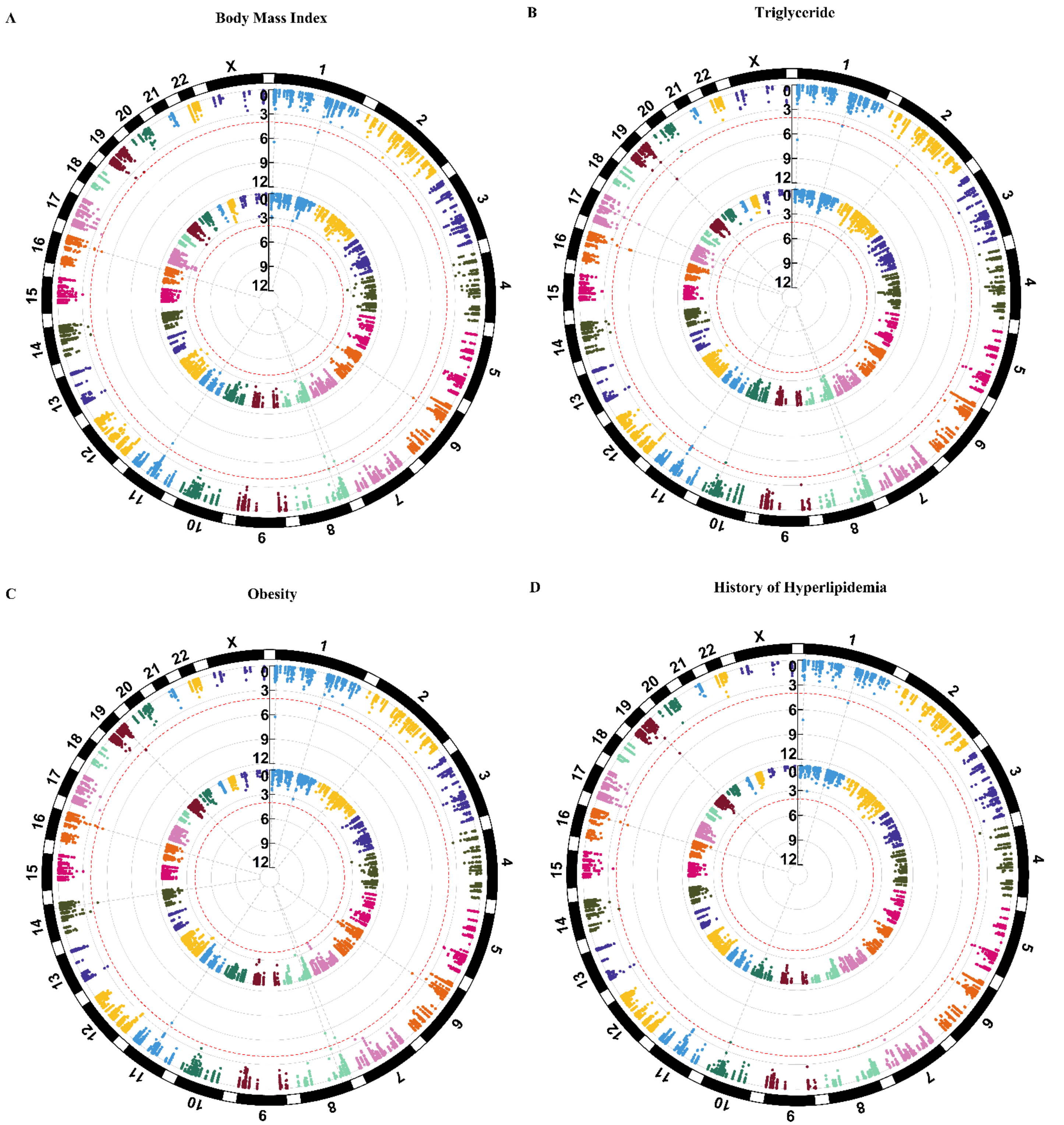

E

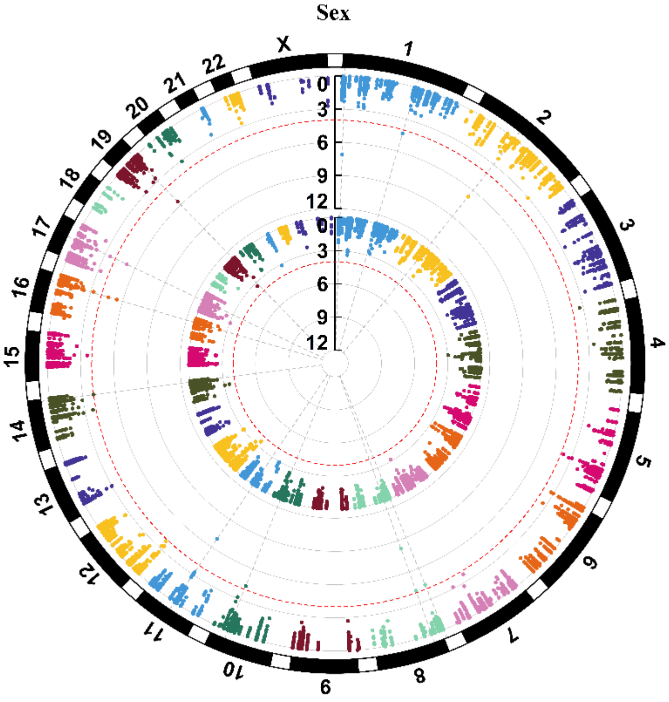

Supplement: Supplementary file 1 — Tables S1–S21 Figure S1–S2 [file JAH3-11-e025245-s001.pdf]
